# Real-world data on clinical response to inflammatory bowel disease biological treatments in patients with concurrent primary sclerosing cholangitis: a case–control study

**DOI:** 10.1093/gastro/goag079

**Published:** 2026-07-30

**Authors:** Robert Tosse, Martin Maibier, Andreas Fischer, Marcel Razpotnik, Konstantinos Kouladouros, Frank Tacke, Michael Sigal, Jonas Wizenty

**Affiliations:** Department of Hepatology and Gastroenterology, Charité-Universitätsmedizin Berlin, Campus Virchow-Klinikum (CVK) and Campus Charité Mitte (CCM), Berlin 13353, Germany; Department of Hepatology and Gastroenterology, Charité-Universitätsmedizin Berlin, Campus Virchow-Klinikum (CVK) and Campus Charité Mitte (CCM), Berlin 13353, Germany; Department of Hepatology and Gastroenterology, Charité-Universitätsmedizin Berlin, Campus Virchow-Klinikum (CVK) and Campus Charité Mitte (CCM), Berlin 13353, Germany; Department of Hepatology and Gastroenterology, Charité-Universitätsmedizin Berlin, Campus Virchow-Klinikum (CVK) and Campus Charité Mitte (CCM), Berlin 13353, Germany; Department of Hepatology and Gastroenterology, Charité-Universitätsmedizin Berlin, Campus Virchow-Klinikum (CVK) and Campus Charité Mitte (CCM), Berlin 13353, Germany; Department of Hepatology and Gastroenterology, Charité-Universitätsmedizin Berlin, Campus Virchow-Klinikum (CVK) and Campus Charité Mitte (CCM), Berlin 13353, Germany; Department of Hepatology and Gastroenterology, Charité-Universitätsmedizin Berlin, Campus Virchow-Klinikum (CVK) and Campus Charité Mitte (CCM), Berlin 13353, Germany; Berlin Institute for Medical Systems Biology (BIMSB), Max Delbrück Center for Molecular Medicine in the Helmholtz Association (MDC), Berlin 13125, Germany; Department of Hepatology and Gastroenterology, Charité-Universitätsmedizin Berlin, Campus Virchow-Klinikum (CVK) and Campus Charité Mitte (CCM), Berlin 13353, Germany; Berlin Institute of Health at Charité-Universitätsmedizin Berlin, BIH Biomedical Innovation Academy, BIH Charité Clinician Scientist Program, Berlin 10178, Germany

**Keywords:** inflammatory bowel disease, primary sclerosing cholangitis, biological therapy, ustekinumab, infliximab, vedolizumab

## Abstract

**Background:**

The intestinal therapy response in patients with primary sclerosing cholangitis-associated inflammatory bowel disease (PSC-IBD) is not well explored. This study compared the intestinal therapy response to biological therapies in patients with PSC and IBD (PSC-IBD group) vs patients with IBD alone (IBD group).

**Methods:**

We performed a case–control study and identified 46 patients in the PSC-IBD group and 180 in the IBD group who were treated with infliximab, vedolizumab, and/or ustekinumab at the IBD outpatient clinic of Charité-Universitätsmedizin Berlin, Campus-Virchow-Klinikum. To account for differences in demographics and disease characteristics, propensity score matching (ratio 1:1) was performed. The primary outcome was clinical therapy response within 20 weeks, defined as remission (partial Mayo Score ≤ 1 or Harvey-Bradshaw-Index < 5) or partial response (decrease of partial Mayo Score ≥ 2 or a decrease of Harvey-Bradshaw-Index > 3). Secondary outcomes included treatment persistence, endoscopic response, decrease in faecal calprotectin, concomitant medication and safety.

**Results:**

After matching, 46 patients per group were analysed (median follow-up: PSC-IBD: 44 months; IBD: 60 months), with a total of 136 treatments administered. The cohort demographics and disease characteristics were balanced between both groups. While clinical response rates did not significantly differ between groups in patients receiving infliximab or vedolizumab, in patients receiving ustekinumab, clinical response rates within the first 20 weeks were significantly lower in the PSC-IBD group (9 of 21, 42.9%) compared with the IBD group (20 of 26, 76.9%; *P *= 0.017). Treatment persistence after 12 months of patients on biological therapy did not differ significantly between groups across all therapies, but a numerically lower persistence rate was observed for ustekinumab in the PSC-IBD group (6 of 21, 28.6%) compared with the IBD group (10 of 22, 45.5%; *P *= 0.252).

**Conclusion:**

Infliximab and vedolizumab appear equally effective for achieving clinical response in patients with PSC-IBD and IBD, while the early clinical response to ustekinumab was significantly lower in patients with PSC-IBD.

## Introduction

Inflammatory bowel diseases (IBD), such as ulcerative colitis (UC) and Crohn’s disease (CD), are immune-mediated, chronic, relapsing, and remitting disorders of the gastrointestinal tract [1]. The global incidence and prevalence have been steadily increasing over the past century and are expected to continue rising in the coming years [2, 3]. Western countries in Europe and North America have the highest prevalence, with up to 750 per 100,000 persons for UC and up to 300 per 100,000 persons for CD [3, 4]. Advanced biological treatments have improved outcomes for patients diagnosed with IBD. However, primary non-response and secondary loss of response remain significant therapeutic challenges [5, 6].

IBD is associated with primary sclerosing cholangitis (PSC) [7, 8]. About 2.16% of the patients with IBD are suffering from PSC (2.47% for UC and 0.96% for CD) [7, 9]. There is an ongoing discussion about whether PSC is an extraintestinal manifestation of IBD or an independent disease [10]. IBD in patients with PSC (PSC-IBD) is characterized by differences in phenotype, for example, the common presence of pancolitis, right-sided disease activity, elevated risk of colorectal cancer, and differences in the immune phenotype [10, 11]. Although several pathophysiological mechanisms have been proposed, the exact pathophysiology of PSC and the link between IBD and PSC remain poorly understood [10, 12].

Despite biological differences in intestinal disease between PSC-IBD and IBD, specific treatment recommendations for PSC-IBD remain lacking due to limited data on therapeutic responses in this rare population [13]. There are only limited studies that have investigated the biological treatment response in patients with PSC-IBD. Most of them primarily analysed the hepatic treatment response (liver-biochemistry and PSC-related complications) rather than the intestinal disease course [14]. A few studies have explored the intestinal therapy response as a secondary outcome, demonstrating clinical response rates of about 50% for infliximab and vedolizumab, and endoscopic response rates of about 30% for both therapies [15–19]. Importantly, to the best of our knowledge, none of these studies directly compared the intestinal therapy response between patients diagnosed with PSC-IBD and those diagnosed with IBD alone. Furthermore, no peer-reviewed studies have explored the intestinal therapy response to ustekinumab in PSC-IBD [13, 20, 21].

Based on these gaps, our study aimed to retrospectively analyse the intestinal therapy response to biological treatments (infliximab, vedolizumab, and ustekinumab) in matched clinical cohorts (PSC-IBD vs IBD alone) using data from a large tertiary gastroenterology and hepatology centre in Germany. Our particular interest was whether PSC-IBD, with a distinct biological profile, influences response to biological therapies with different mechanisms of action.

## Materials and methods

### Study design

This single-centre case–control study compared the intestinal therapy response to IBD treatment with infliximab, vedolizumab, and ustekinumab between patients with PSC-IBD and IBD alone. The study was approved by the ethical committee of Charité-Universitätsmedizin Berlin (EA4/223/23). According to the ethical committee, an informed consent was not required from patients. This study respected the institutional guidelines of good scientific practice and followed the STROBE guideline for case–control studies [22].

### Study population

We identified patients who consulted the IBD outpatient clinic at Charité-Universitätsmedizin Berlin, Campus Virchow-Klinikum, between January 2020 and November 2023. Patient charts were retrospectively reviewed, and we analysed clinical courses of patients aged ≥ 18 years (as of November 2023) with infliximab, vedolizumab, and/or ustekinumab therapy. Both IBD and PSC had to be diagnosed based on internationally accepted diagnostic criteria [23, 24]. Patient eligibility was confirmed by two independent investigators (R.T. and J.W.).

**Figure 1:**
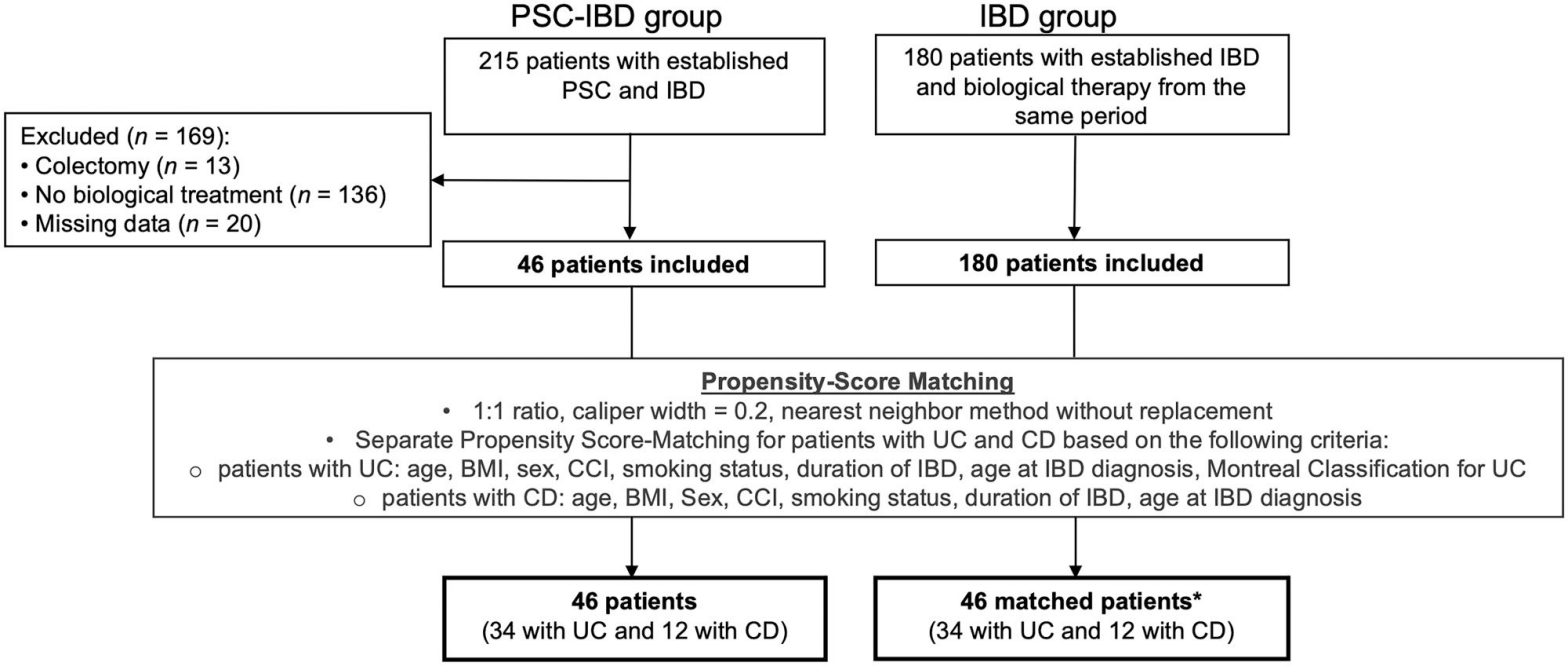
Patient selection and propensity score-matching. PSC, primary sclerosing cholangitis; IBD, inflammatory bowel disease; UC, ulcerative colitis; CD: Crohn’s disease; BMI, body mass index; CCI, Charlson Comorbidity index (Modification Quan *et al*.). *Three patients replaced due to missing data (selection of other patients based on the propensity score).

Patients who underwent colectomy prior to the biological treatment or had missing follow-up data (e.g. clinical score values) were excluded. After applying inclusion and exclusion criteria, we included 46 patients in the PSC-IBD group and 180 patients from the same period in the IBD group (Fig. 1). In the IBD outpatient clinic, the indication for biological treatment was determined by a specialized gastroenterologist. Patients were seen regularly every 3 to 6 months, and all biological treatments were given on-label. All therapy trajectories with infliximab, vedolizumab, and ustekinumab given to these patients in their history were analysed, except therapies administered within clinical trials, therapies initiated after August 2023, and re-induction therapies.

### Data collection

We collected demographic data and disease-specific data on IBD and PSC, including: age, sex, smoking status, body mass index (BMI), comorbidities, age at IBD diagnosis, duration of IBD, disease location (Montreal Classification), extraintestinal manifestations, IBD treatment, IBD-related surgeries, IBD-related complications, age at PSC diagnosis, duration of PSC disease, PSC overlap syndrome, PSC-related complications, data on PSC-related hepatic cirrhosis, and history of hepatic transplantation with related complications. We calculated the Charlson Comorbidity Index (CCI) as modified by Quan *et al.*, without including PSC and related complications [25]. We measured clinical response to therapy by retrospectively scoring the partial Mayo Score (pMS) for patients diagnosed with UC and the Harvey-Bradshaw Index (HBI) for patients diagnosed with CD using patient charts [26, 27]. We did not score the pMS or HBI if any of the components: stool frequency, rectal bleeding, general well-being/physician’s global assessment or abdominal pain were unavailable in the clinical chart. For the components abdominal mass and complications of HBI a score of zero was assigned when no positive value was documented. The endoscopic response was analysed using colonoscopy and histology reports and scored as Mayo Endoscopic Score for UC and categorized as none, mild, medium, or severe inflammation for CD [28]. Faecal calprotectin (FC) values were retrieved from patient charts [29]. The data were extracted retrospectively from standardized electronic medical records after November 2023, using identical procedures for both groups.

### Propensity score matching

Propensity score matching was employed to account for differences in cohort demographics and disease characteristics between the study groups. Separate matching procedures were conducted for patients diagnosed with UC and CD. A 1:1 ratio and the nearest neighbour method without replacement were applied, using a calliper width of 0.2 [30]. Patients with UC were matched by age, sex, BMI, smoking status, modified Charlson Comorbidity Index (CCI) , IBD duration, age at IBD diagnosis, and disease location (Montreal Classification). Patients with CD were matched by age, sex, BMI, smoking status, CCI, IBD duration, and age at IBD diagnosis. In the IBD group, three matched patients with missing data were replaced by patients with the next nearest propensity score (Fig. 1). To control the matching quality, we calculated the standardized mean difference (SMD) for the matching variables. A SMD < 0.1 was considered as well balanced and a SMD < 0.2 as balanced [31].

### Study outcomes

The primary outcome was clinical therapy response within the first 20 weeks of therapy, defined as remission (pMS ≤ 1 or HBI < 5) or partial response (decrease of pMS ≥ 2 or a decrease of HBI by > 3) [28]. Patients who did not meet these criteria were classified as non-responders. We analysed the primary outcome within the following subgroups: patients diagnosed with UC, subgroups of patients based on therapy lines, patients with liver cirrhosis and liver transplantation (pre-/posttransplant) within the PSC-IBD group. The subgroup of patients diagnosed with CD was not analysed due to a small sample size. We conducted a head-to-head comparison between infliximab, vedolizumab, and ustekinumab treatment in patients with PSC-IBD.

Secondary outcomes were treatment persistence at 12 months and over the entire study period assessed using Kaplan-Meier curves. We calculated the mean ± standard deviation (SD) therapy duration for the respective biological treatment among patients who completed their therapy during the study period. We analysed explorative data on endoscopic response (mucosal healing defined as absent or mild inflammation in colonoscopy and histology) and decreased levels of FC (FC < 150 µg/g or a decrease by ≥ 50% compared with baseline) in patients who underwent colonoscopy or FC assessment [28, 32]. Further secondary analyses were concomitant medication use, including corticosteroid use within the first 20 weeks of therapy, and safety.

### Statistical analyses

Categorical data are presented as frequencies and percentages; continuous data are shown as median and interquartile range (IQR) or mean and SD. Group differences in cohort demographics and disease characteristics were compared using the Chi-square test or Fisher’s Exact test for categorical data and the Mann-Whitney *U*-test or the independent *t*-test for continuous data. Group differences in outcomes were compared for each medication between the PSC-IBD group and the IBD group. To analyse the primary outcome, we conducted a Chi-square test or Fisher’s Exact test to compare the proportions of patients achieving clinical remission or partial response vs no response for each therapy. To investigate whether the therapy line influenced the primary outcome in patients treated with ustekinumab, we used the binary logistic regression to conduct a univariate analysis and a multivariate analysis (adjusted for therapy line). We analysed the subgroups, the head-to-head comparison, and the secondary outcomes (treatment persistence at 12 months, endoscopic response and decreased levels of FC) using Chi-square test or Fisher’s Exact test. The Log-Rank test was used to analyse treatment persistence in the Kaplan-Meier curves. A *P-*value < 0.05 was considered statistically significant. All statistical analyses were performed using SPSS v29.0.1.1 and Excel v2603. Graphs were designed utilizing GraphPad Prism v9.5.1.

## Results

### Study cohort

After propensity score matching, 92 patients (46 per group) were analysed in this study. In both the PSC-IBD group and the IBD group, 34 patients (73.9%) were diagnosed with UC and 12 patients (26.1%) with CD. Median age was 37.5 years (IQR 32–44 years) in the PSC-IBD group and 37 years (IQR 30–48 years) in the IBD group (*P *= 0.815, SMD = 0.01). Most patients with UC in both groups had a pancolitis [PSC-IBD: 31 of 33 (93.9%) vs IBD group: 26 of 32 (81.3%); *P *= 0.150, SMD = 0.38]. Among the patients with CD, ileocolonic disease location was the most common [PSC-IBD: 6 of 11 (54.5%) vs IBD group: 7 of 12 (58.3%)], followed by colonic disease location [PSC-IBD: 4 of 11 (36.4%) vs IBD group: 1 of 12 (8.3%)] and ileal disease location [PSC-IBD: 1 of 11 (9.1%) vs IBD group: 4 of 12 (33.3%)] (*P *= 0.207, SMD = 0.08). 77 patients [PSC-IBD: 39 of 46 (84.8%) vs IBD group: 38 of 46 (82.6%); *P *= 0.778] had no IBD-related surgery. Liver cirrhosis was diagnosed in 15 patients (32.6%) within the PSC-IBD group (Supplementary Table S1). The cohort demographics and disease characteristics were mostly balanced between the PSC-IBD group and the IBD group (Fig. 1, Table 1, Supplementary Table S2 and Fig. S1). The cohort demographics and disease characteristics for each treatment subgroup can be found in Supplementary Table S3.

**Table 1:** Cohort demographics and disease characteristics among original and matched cohort.

Variables	Original cohort					Matched cohort				
	PSC-IBD (*n *= 46)	IBD (*n *= 180)	*t*/MWU/Chi2/FE	*P*-value	SMD	PSC-IBD (*n *= 46)	IBD (*n *= 46)	t/MWU/Chi2/FE	*P*-value	SMD
Age, years	37.5 (32–44)	41 (33–54.5)	3550.0	0.136	0.29	37.5 (32–44)	37 (30–48)	1028.0	0.815	0.01
Female	15 (32.6)	79 (43.9)	1.919	0.166	0.23	15 (32.6)	22 (47.8)	2.215	0.137	0.31
Follow-up time, months	44 (18–62)	60 (28.5–100.5)	2994.0	0.004	–	44 (18–62)	55.5 (24–81)	856.5	0.116	–
Body mass index, kg/m^2^	22.6 (20.9–24.6)	24.7 (22.3–28.1)	2490.5	< 0.001	0.55	22.6 (20.9–24.6)	23.0 (19.3–26.4)	947.5	0.733	0.08
Smoker	9 (20.0)	68 (44.4)	8.743	0.003	0.54	9 (20.0)	14 (34.1)	2.191	0.139	0.32
Dead during follow-up	4 (8.7)	0 (0.0)	–	0.002	–	4 (8.7)	0 (0.0)	–	0.117	–
CCI, mean ± standard deviation	0.6 ± 1.1	0.6 ± 1.3	0.062	0.951	0.01	0.6 ± 1.1	0.5 ± 1.0	-0.398	0.766	0.10
IBD-subtype										
UC	34 (73.9)	130 (72.2)	0.053	0.819	0.04	34 (73.9)	34 (73.9)	0.000	1.000	0.00
CD	12 (26.1)	50 (27.8)				12 (26.1)	12 (26.1)			
Age at initial diagnosis, years	21 (16–27)	25 (20–35)	2907.5	0.002	0.50	21 (16–27)	21 (18–29)	959.5	0.441	0.18
Duration of IBD, years	15 (11–24)	13 (7–20)	4588.5	0.231	0.13	15 (11–24)	11 (6–20)	871.0	0.144	0.17
Montreal Classification for UC			24.158	< 0.001	1.09^a^			3.150	0.150	0.38^a^
Proctitis	1 (3.0)	5 (4.3)				1 (3.0)	1 (3.1)			
Left-sided	1 (3.0)	52 (44.4)				1 (3.0)	5 (15.6)			
Pancolitis	31 (93.9)	60 (51.3)				31 (93.9)	26 (81.3)			
Montreal Classification for CD										
Ileal	1 (9.1)	12 (24.0)	6.368	0.074	0.15^b^	1 (9.1)	4 (33.3)	4.372	0.207	0.08^b^
Colon	4 (36.4)	7 (14.0)				4 (36.4)	1 (8.3)			
Ileocolon	6 (54.5)	31 (62.0)				6 (54.5)	7 (58.3)			
Upper GI	4 (36.4)	12 (24.0)	4.115	0.139	–	4 (36.4)	3 (25.0)	1.337	0.667	–
Extraintestinal manifestations	13 (28.3)	39 (21.7)	1.032	0.310	–	13 (28.3)	12 (26.1)	0.055	0.815	–
Surgical IBD-Therapy	7 (15.2)	39 (21.8)	0.971	0.324	–	7 (15.2)	8 (17.4)	0.080	0.778	–

Data are presented as median (interquartile range) or number (percentage), except CCI.

CCI, Charlson Comorbidity Index (Modification Quan *et al.*); IBD, inflammatory bowel disease; UC, ulcerative colitis; CD, Crohn’s disease; *t: t*-test; MWU: Mann-Whitney-*U*-test; Chi2: Chi-Square-test; FE, Fisher’s Exact-test; PSC-IBD, primary sclerosing cholangitis associated inflammatory bowel disease; SMD, standardized mean difference.

SMD calculated for matching variables only: < 0.1 well balanced, < 0.2 balanced, > 0.2 potential bias.

a SMD calculated pancolitis vs no pancolitis.

b SMD calculated ileocolon vs no ileocolon.

In total, 136 therapies were administered. Among these, 39 patients received infliximab (PSC-IBD: *n *= 19; IBD group: *n *= 20), 50 patients were treated with vedolizumab (PSC-IBD: *n *= 28; IBD group: *n *= 22), and 47 patients received ustekinumab (PSC-IBD: *n *= 21; IBD group: *n *= 26). Among patients who received infliximab, 9 of 19 patients (47.4%) in the PSC-IBD group and 13 of 20 patients (65.0%) in the IBD group received no concomitant IBD medication. In the PSC-IBD group, 14 of 19 patients (73.6%) and in the IBD group, 18 of 20 patients (90.0%) received infliximab as their first or second biological treatment for IBD. In the group of patients who received vedolizumab, 10 of 28 patients (35.7%) in the PSC-IBD group and 12 of 22 patients (54.6%) in the IBD group received no additional IBD medication. Vedolizumab was used as the first or second biological treatment for IBD in 25 of 28 patients (89.2%) of the PSC-IBD group and in 20 of 22 patients (90.9%) of the IBD group. Among patients who received ustekinumab, 12 of 21 patients (57.1%) in the PSC-IBD group and 22 of 26 patients (84.6%) in the IBD group received no additional IBD medication. In the PSC-IBD group, 11 of 21 patients (52.4%) and in the IBD group, 15 of 26 patients (57.7%) received ustekinumab as first or second biological treatment for IBD. Notably, under real-world conditions, ustekinumab was used as first biological treatment only in 2 of 21 patients (9.5%) of the PSC-IBD group compared with 9 of 26 patients (34.6%) in the IBD group. Overall, there were no statistically significant differences in concomitant medication use and line of therapy across all three therapies between the groups. The most frequently used concomitant medication was mesalamine (Supplementary Table S4).

### Primary outcome

#### All patients

**Figure 2:**
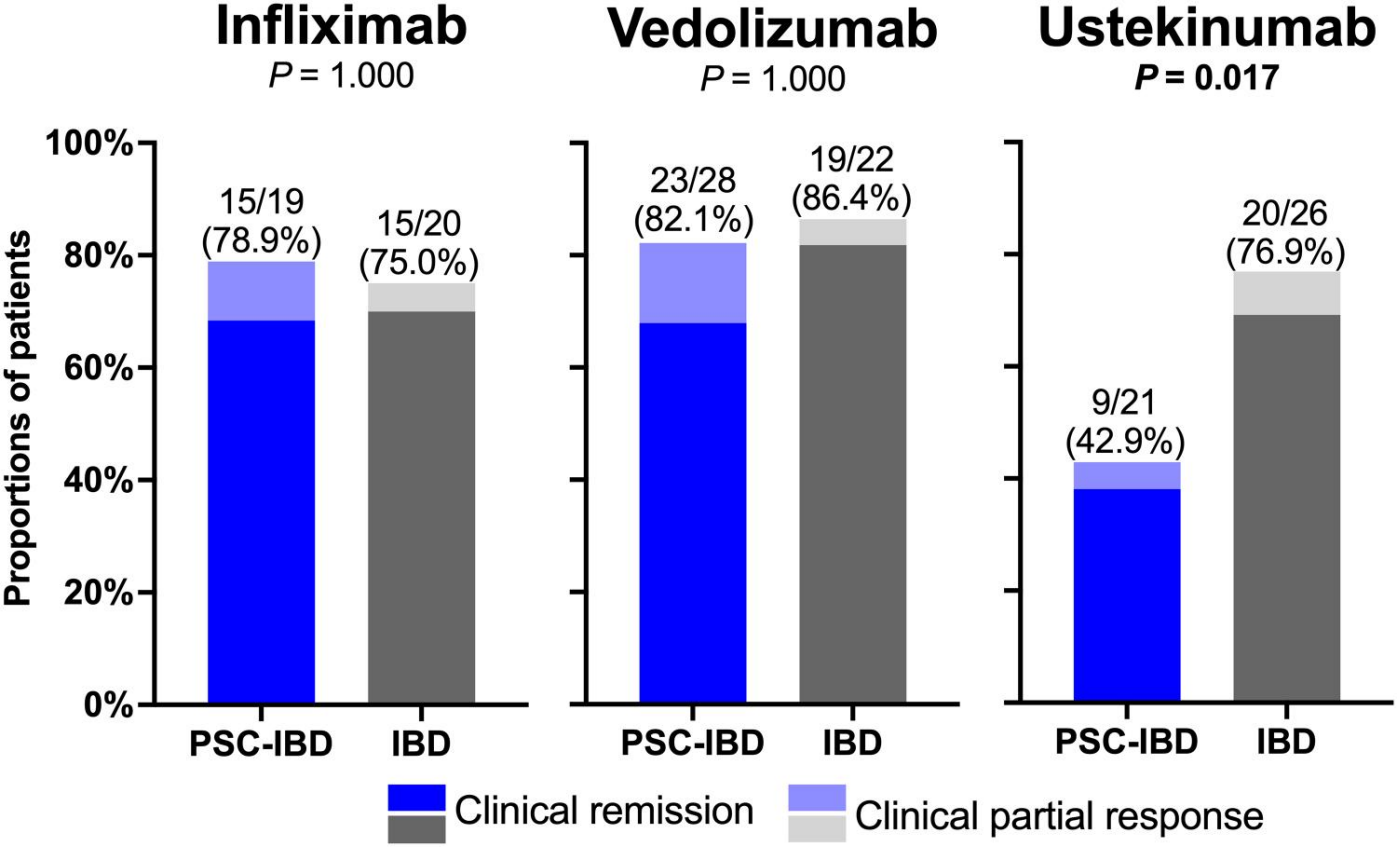
Clinical response within 20 weeks between the PSC-IBD group and the IBD group for patients treated with infliximab, vedolizumab, and ustekinumab. PSC, primary sclerosing cholangitis; IBD, inflammatory bowel disease.

The clinical response rates within the first 20 weeks of therapy did not significantly differ between the PSC-IBD group and the IBD group in patients receiving infliximab and vedolizumab. Clinical response in patients treated with infliximab was achieved by 15 of 19 patients (78.9%) in the PSC-IBD group [clinical remission: 13 of 19 patients (68.4%); partial response: 2 of 19 patients (10.5%)] and 15 of 20 patients (75.0%) in the IBD group [clinical remission: 14 of 20 patients (70.0%); partial response: 1 of 20 patients (5.0%)], *P *= 1.00. In patients receiving vedolizumab, clinical response was achieved by 23 of 28 patients (82.1%) in the PSC-IBD group [clinical remission: 19 of 28 patients (67.9%); partial response: 4 of 28 patients (14.3%)] and 19 of 22 patients (86.4%) in the IBD group [clinical remission: 18 of 22 patients (81.8%); partial response: 1 of 22 patients (4.6%)], *P *= 1.00 (Fig. 2 and Supplementary Table S5). Contrarily, in patients treated with ustekinumab, proportions of patients with clinical response within the first 20 weeks of therapy were significantly lower in the PSC-IBD group compared with the IBD group. Clinical response was achieved by 9 of 21 patients (42.9%) in the PSC-IBD group [clinical remission: 8 of 21 patients (38.1%); partial response: 1 of 21 patients (4.8%)] and 20 of 26 patients (76.9%) in the IBD group [clinical remission: 18 of 26 patients (69.2%); partial response: 2 of 26 patients (7.7%)], *P *= 0.017 (Fig. 2 and Supplementary Table S5). The proportion of patients who used corticosteroids within the first 20 weeks of therapy did not differ significantly between groups for patients receiving infliximab [PSC-IBD: 9 of 17 (52.9%) vs IBD group: 11 of 19 (57.9%), *P *= 0.765], vedolizumab [PSC-IBD: 12 of 22 (54.5%) vs IBD group: 10 of 21 (47.6%), *P *= 0.650] and ustekinumab [PSC-IBD: 11 of 20 (55.0%) vs IBD group: 15 of 26 (57.7%), *P *= 0.855] (Supplementary Table S4).

#### Subgroup of patients with UC

To explore the clinical response rates specifically in patients with UC, we performed a subgroup analysis of patients diagnosed with UC for the primary outcome. As in the main analysis, in patients treated with ustekinumab, the rate of clinical response was lower in the PSC-IBD group, although the difference did not reach statistical significance. Clinical response was achieved by 5 of 14 patients (35.7%) in the PSC-IBD group [clinical remission: 4 of 14 patients (28.6%); partial response: 1 of 14 patients (7.1%)] and 11 of 16 patients (68.8%) in the IBD group [clinical remission: 10 of 16 patients (62.5%); partial response: 1 of 16 patients (6.3%)], *P *= 0.070. No statistically significant differences were seen among patients treated with infliximab and vedolizumab (Supplementary Tables S6 and S7).

#### Subgroup of patients based on therapy line

As only 2 of 21 patients (9.5%) in the PSC-IBD group received ustekinumab as first-line therapy vs 9 of 26 patients (34.6%) in the IBD group, we analysed the clinical response rates in bio-naïve and bio-exposed patients separately. Consistent with the main analysis, the clinical response rates to infliximab and vedolizumab did not significantly differ between both groups in either bio-naïve or bio-exposed patients. Among bio-exposed patients treated with ustekinumab, clinical response (clinical remission or partial response) was less frequent in the PSC-IBD group [8 of 19 patients (41.2%)] compared with the IBD group [11 of 17 patients (64.8%)], although this difference did not reach statistical significance (*P *= 0.175) (Supplementary Table S8). To further investigate whether the therapy line influenced the primary outcome in patients treated with ustekinumab, we conducted a binary logistic regression for these patients. Consistent with the main analysis, the univariate analysis showed significantly lower odds of clinical response to ustekinumab in the PSC-IBD group (odds ratio 0.225, 95% confidence interval: 0.064–0.791, *P *= 0.020). This finding remained significant in the multivariate analysis adjusted for therapy line (odds ratio 0.228, 95% confidence interval: 0.053–0.970, *P *= 0.045). We conducted a separate sensitivity analysis with patients receiving ustekinumab as first-, second-, or third-line therapy. Consistent with the main analysis, a significantly lower clinical response rate was observed in the PSC-IBD group [PSC-IBD: 7 of 16 (43.8%) vs IBD group: 18 of 23 (78.3%), *P *= 0.027] (Supplementary Table S8).

#### Subgroup of patients with liver cirrhosis and liver transplantation within the PSC-IBD group

A total of 15 patients in the PSC-IBD group had a diagnosed liver cirrhosis. In this patient population, 8 trajectories of infliximab, 11 trajectories of vedolizumab, and 8 trajectories of ustekinumab were analysed. The primary outcome did not differ between patients with and without liver cirrhosis within the PSC-IBD group for all three biologic therapies (Supplementary Table S1). The subgroup of patients after liver transplantation within the PSC-IBD group was small (trajectories: 1 infliximab, 3 vedolizumab, 1 ustekinumab) and therefore not analysed.

#### Head-to-head comparison in patients with PSC-IBD

The clinical response rates within the first 20 weeks of therapy did not significantly differ between patients receiving infliximab and vedolizumab (*P *= 1.0) within the PSC-IBD group. In patients treated with ustekinumab, proportions of patients with clinical response within the first 20 weeks of therapy were significantly lower compared with patients receiving infliximab (*P *= 0.027) or vedolizumab (*P *= 0.007) within the PSC-IBD group (Supplementary Table S9 and Fig. S2).

### Secondary outcomes

#### Treatment persistence

**Figure 3:**
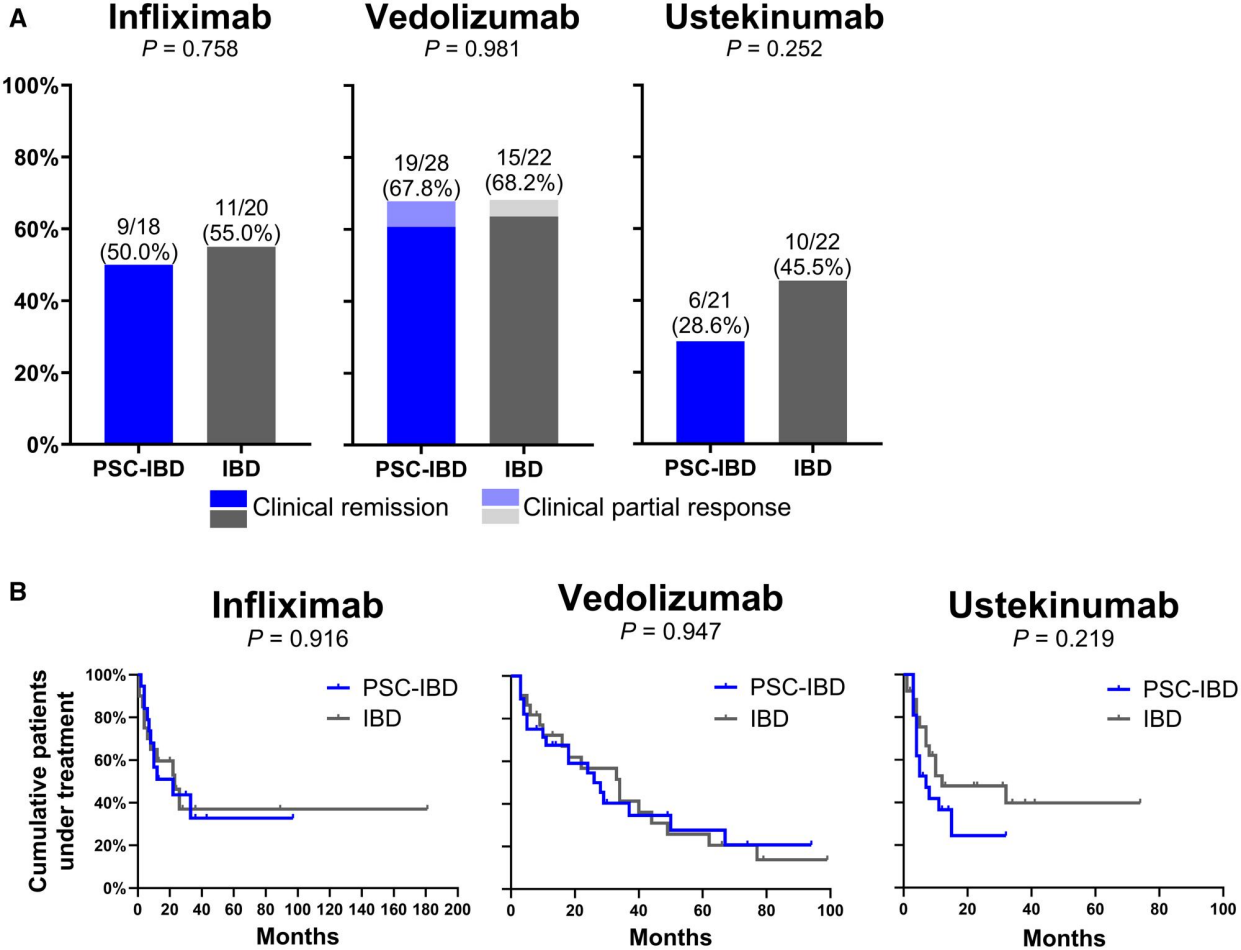
Clinical response between the PSC-IBD group and the IBD group for patients treated with infliximab, vedolizumab, and ustekinumab. (A) Clinical response after 12 months of therapy: percentage of patients who have continued therapy. (B) Kaplan-Meier Curves. Patients who discontinued therapy due to other reasons than loss of response (e.g. adverse drug reactions or patient request) and patients with ongoing therapy at the end of the study period were censored. PSC, primary sclerosing cholangitis; IBD, inflammatory bowel disease.

After 12 months of therapy the proportion of patients with clinical response in the PSC-IBD group compared with the IBD group did not significantly differ in patients receiving infliximab [PSC-IBD: 9 of 18 (50.0%) vs IBD group: 11 of 20 (55.0%), *P *= 0.758], vedolizumab [PSC-IBD: 19 of 28 (67.8%) vs IBD group: 15 of 22 (68.2%), *P *= 0.981] and ustekinumab [PSC-IBD: 6 of 21 (28.6%) vs IBD group: 10 of 22 (45.5%), *P *= 0.252] (Fig. 3A). We found no statistically significant differences in treatment persistence in the Kaplan-Meier curves between the PSC-IBD group and the IBD group for patients treated with infliximab (*P *= 0.916), vedolizumab (*P *= 0.947), and ustekinumab (*P *= 0.219) throughout the whole observation period (Fig. 3B). The reasons for treatment discontinuation are described in Supplementary Table S4.

The therapy durations were for infliximab 14 ± 11 months in the PSC-IBD group and 18 ± 22 months in the IBD group, for vedolizumab 20 ± 17 months in the PSC-IBD group and 26 ± 22 months in the IBD group, and for ustekinumab 7 ± 4 months in the PSC-IBD group and 10 ± 10 months in the IBD group (Supplementary Table S4).

Although statistical significance was not reached, a trend toward shorter treatment persistence was most pronounced for ustekinumab, where 12-month persistence in the PSC-IBD group was 16.9% age points lower than in the IBD group, consistent with an early separation of the Kaplan-Meier curves between groups.

#### Endoscopic response

**Figure 4:**
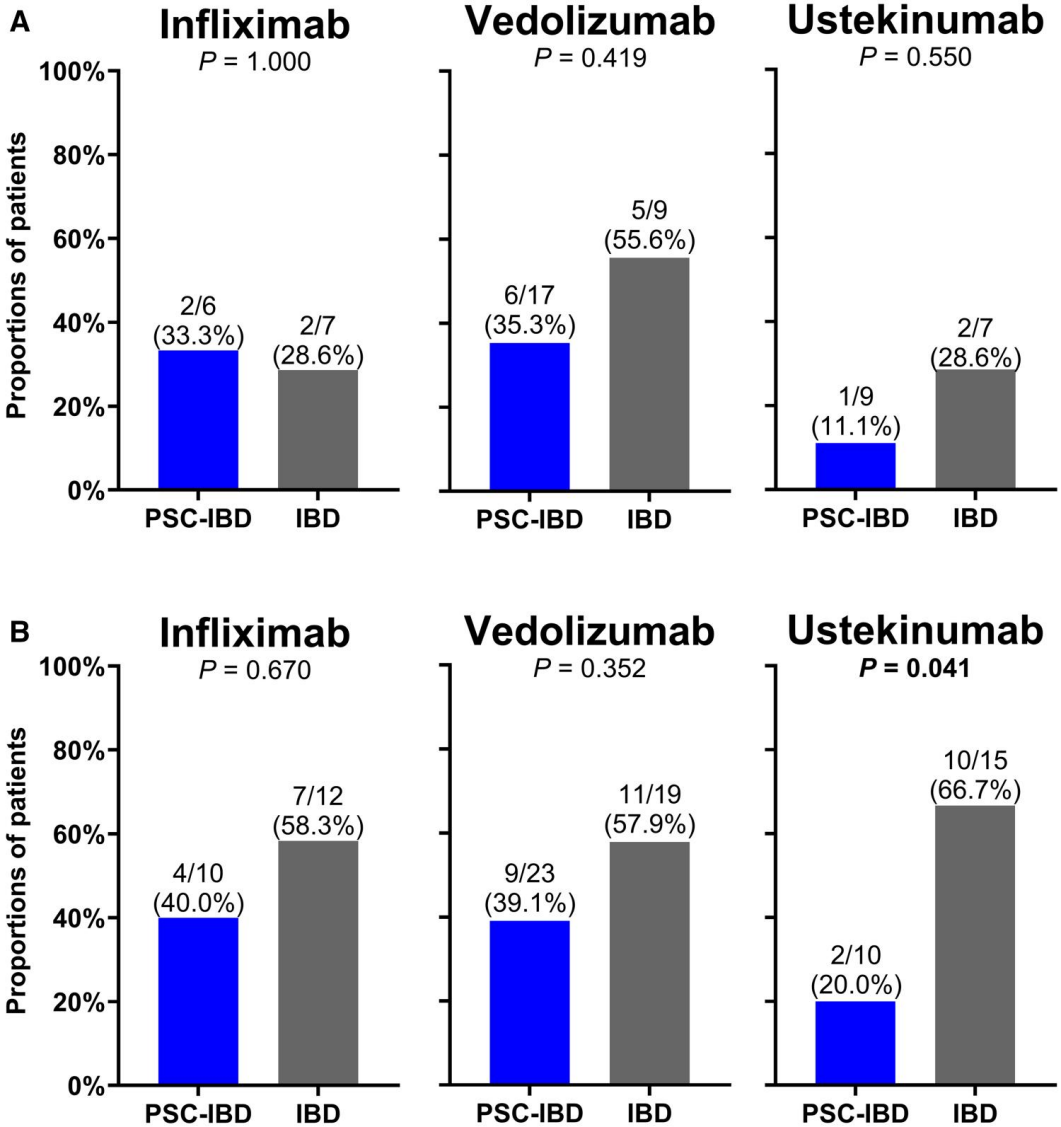
Mucosal healing between the PSC-IBD group and the IBD group for patients treated with infliximab, vedolizumab, and ustekinumab. (A) Mucosal healing during the first 12 months of therapy. (B) Mucosal healing over the whole study period. Patients without colonoscopy and histology were not included. PSC, primary sclerosing cholangitis; IBD, inflammatory bowel disease.

A minor subset of patients underwent colonoscopy. This exploratory data demonstrated no difference after 12 months [PSC-IBD: 1 of 9 (11.1%) vs IBD group: 2 of 7 (28.6%), *P *= 0.550; Fig. 4A], but a lower mucosal healing rate over the whole study period [PSC-IBD: 2 of 10 (20.0%) vs IBD group: 10 of 15 (66.7%), *P *= 0.041; Fig. 4B] in the PSC-IBD group of patients treated with ustekinumab compared with the IBD group. No differences were observed for infliximab and vedolizumab (Fig. 4 and Supplementary Table S5).

#### Faecal calprotectin decrease

**Figure 5:**
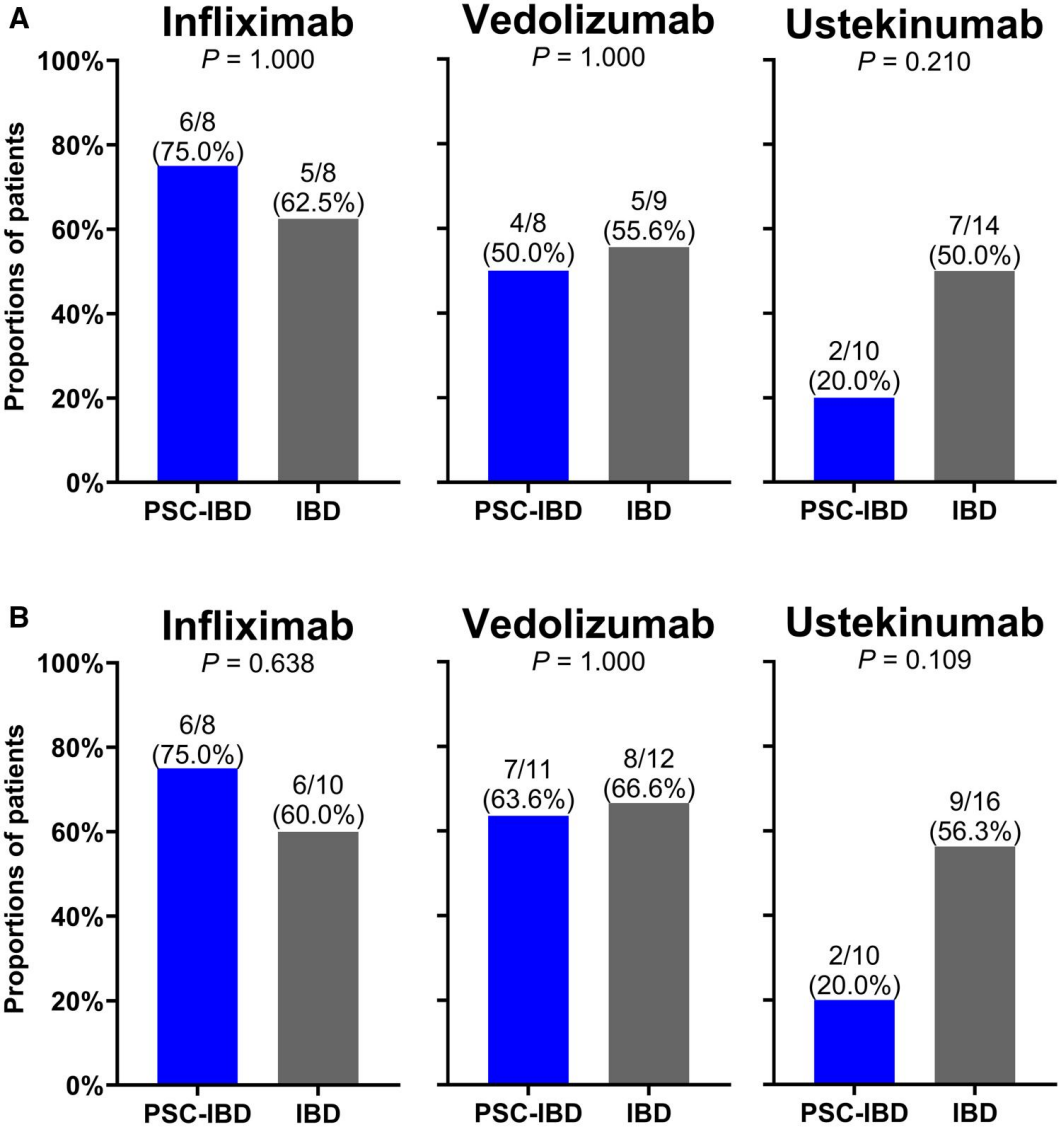
FC decrease between the PSC-IBD group and the IBD group for patients treated with infliximab, vedolizumab, and ustekinumab. (A) Decreased levels of faecal calprotectin (FC < 150 µg/g or decreased by more than 50%) during the first 12 months of therapy. (B) Decreased levels of FC over the whole study period. Patients without available FC values were not included. PSC, primary sclerosing cholangitis; IBD, inflammatory bowel disease; FC, faecal calprotectin.

Despite the limited sample size, patients in the PSC-IBD group treated with ustekinumab showed a trend towards lower rates of faecal calprotectin (FC) decrease, both at 12 months [PSC-IBD: 2 of 10 (20.0%) vs IBD group: 7 of 14 (50.0%), *P *= 0.210; Fig. 5A] and over the full study period [PSC-IBD: 2 of 10 (20.0%) vs IBD group: 9 of 16 (56.3%), *P *= 0.109; Fig. 5B]. No trends in patients treated with infliximab and vedolizumab were observed between the PSC-IBD group and the IBD group (Fig. 5 and Supplementary Table S5).

#### Safety

In total, the rate of adverse events leading to therapy discontinuation was 7.35% (10 of 136) across all three biological treatments and was not different between groups [PSC-IBD: 4 of 68 (5.9%) vs IBD group: 6 of 68 (8.8%), *P *= 0.511]. Discontinuation occurred in 7 of 39 (17.9%) patients treated with infliximab [PSC-IBD: 2 of 19 (10.5%) vs IBD group: 5 of 20 (25.0%)], in 1 of 50 (2.0%) patients treated with vedolizumab [PSC-IBD: 1 of 28 (3.6%) vs IBD group: 0 of 22 (0%)] and among 2 of 47 (4.3%) patients treated with ustekinumab [PSC-IBD: 1 of 21 (4.8%) vs IBD group: 1 of 26 (3.9%)]. Notably, therapy discontinuation due to infectious complications such as ascending cholangitis (1 PSC-IBD patient per biological therapy) were extremely rare (Supplementary Table S4).

## Discussion

This study found that infliximab and vedolizumab are equally effective in achieving intestinal clinical response in patients with PSC-IBD compared with patients with IBD alone. In contrast, the efficacy of ustekinumab for achieving intestinal clinical response within 20 weeks was significantly lower in patients with PSC-IBD. Consistent with the clinical findings, mucosal healing and reduction in FC levels were also less frequent in patients with PSC-IBD receiving ustekinumab, although the reduction in FC levels did not reach statistical significance. Treatment persistence did not significantly differ between patients with PSC-IBD and IBD alone receiving ustekinumab, neither at 12 months nor across the entire study period; however, a consistent trend toward earlier discontinuation in the PSC-IBD group was observed. In a subgroup analysis, no differences in primary outcome between patients with and without liver cirrhosis were detected.

Real-world response rates in our IBD group were relatively high compared with the 30%–60% reported in previous reviews and meta-analyses [5]. For instance, Schreiber *et al.* described in a meta-analysis a clinical response rate to vedolizumab of 56% (95% CI: 50%–62%) after 14 weeks and 52% (95% CI: 37%–56%) after 12 months in UC, and 58% (95% CI: 51%–64%) after 14 weeks and 40% (95% CI: 29%–52%) after 12 months in CD [33]. These differences could be due to our retrospective study design, including retrospective scoring of pMS and HBI.

A meta-analysis by Shah *et al.* investigated the role of TNF-α inhibitors (infliximab and adalimumab) and vedolizumab in patients with PSC-IBD across four studies [15]. One study explored TNF-α inhibitors [16] and three studies explored vedolizumab [17–19]. The reported overall clinical response was 47% (95% CI: 39.6%–54.5%) with similar results for TNF-α inhibitors and vedolizumab. The reported overall endoscopic response rate was 31.2% (95% CI: 23.8%–39.7%), with vedolizumab showing higher response rates (46.3%; 95% CI: 32.2%–61.1%) than TNF-alpha inhibitors (23.2%; 95% CI: 15.8%–32.7%) [15]. Hedin *et al.* explored the intestinal response after 12 months in patients with PSC-IBD receiving TNF-α inhibitors (infliximab and adalimumab). Therapy response was defined as endoscopic or clinical response, and remission was defined as endoscopic or clinical remission, according to the physician’s assessment. Response was achieved in 48% (50 of 104 patients), and remission was achieved in 23% (22 of 95 patients) after 3 months of therapy [16]. The role of vedolizumab was explored by Caron *et al.*, Christensen *et al.*, and Lynch *et al.* [17–19]. Caron *et al.* assessed 54 patients with PSC-IBD treated with vedolizumab at 54 weeks. Among the 33 patients with UC, 50% achieved steroid-free clinical remission (pMS < 3 with stool frequency and rectal bleeding score of ≤ 1). 27 of these patients underwent endoscopy, with 11 achieving mucosal healing (Mayo Endoscopic Subscore 0 or 1). In 21 patients with CD, 46% achieved steroid-free clinical remission (HBI ≤ 4), and 7 patients underwent endoscopy, with 42.9% (3 of 7) achieving mucosal healing [17]. Christensen *et al.* analysed 25 patients with clinically active PSC-IBD treated with vedolizumab. Clinical remission rates at week 30 based on Simple Clinical Colitis Activity Index ≤ 2 or HBI < 4 were 29% (4 of 14 patients) in patients with UC and 55% (6 of 11 patients) in patients with CD. Endoscopic response [UC: 29% (2 of 7), CD 33% (2 of 6)] and mucosal healing [UC: 14% (1 of 7), CD: 0% (0 of 6)] were slightly lower in this study [18]. Lynch *et al.,* however, reported a higher endoscopic response rate of 56.8% (42 of 74 patients), defined as improved endoscopic classification determined by the treating physician, in patients with PSC-IBD treated with vedolizumab [19]. Overall, these reports align with our finding that infliximab and vedolizumab are effective for achieving clinical response in patients with PSC-IBD, although our response rates were slightly higher. Notably, endoscopic response rates between 29% and 57% were reported [17–19]. This indicates that clinical, endoscopic, and histological responses do not strongly correlate in patients with PSC-IBD [10, 34, 35].

Our data indicate that ustekinumab is less efficient in patients with PSC-IBD compared with patients with IBD alone. The role of ustekinumab has not been extensively explored; only data from two conference abstracts are available [20, 21]. Sarras *et al.* showed in 20 patients with PSC-IBD receiving ustekinumab 0% endoscopic response [20]. An abstract from Al-Shakhshir *et al.* investigated the efficacy of anti-IL-23 agents in 66 patients with PSC-IBD, with 62 patients treated with ustekinumab [21]. The response rates were relatively low (15% at 6 months, 20% at 12 months and 21% at 24 months) and only 3 patients (4.5%) achieved remission defined by Mayo colitis score or FC values. These abstracts support our findings and indicate reduced efficacy of ustekinumab. While ustekinumab targets IL-12 and IL-23, a new generation of more specific anti-IL-23 inhibitors has been recently approved for the treatment of UC and CD [36, 37]. It will be important to explore response rates to these compounds in patients with PSC-IBD. Furthermore, there are other advanced therapies like small molecules, which have not been investigated in patients with PSC-IBD yet [38, 39].

The exact pathophysiology of PSC-IBD is not understood today. Nevertheless, there are pathophysiological differences between PSC-IBD and IBD alone [10, 12]. The gut microbiota is different in patients with PSC compared with healthy controls independent of a concurrent IBD and studies have also found differences in gut microbiota between PSC-IBD and IBD alone [10]. Leibovitzh *et al.* showed a reduced microbial gene richness in patients with PSC-IBD compared with IBD alone, for example, PSC-associated *Klebsiella pneumoniae* or enrichment of *Enterococcus*, *Fusobacterium*, or *Veillonella* species [12, 40]. These differences may lead to inflammation mechanisms that potentially bypass the IL-12/IL-23 pathways.

Also, PSC-IBD and IBD alone contain a different immunological profile [10, 12, 41]. Gwela *et al.* observed a decreased frequency of Th1 cells and an increased frequency of IFN-γ producing T-cells in patients with PSC-IBD compared with IBD alone, whereas Th17 cells were increased in both entities [41]. The decrease of Th1 cells, which are part of the IL-12 pathway, could be a factor explaining why anti-IL-12 is less effective in PSC-IBD [42]. One recent hypothesis linking PSC and IBD is an aberrant trafficking of gut-primed lymphocytes reaching the liver [10, 43]. This phenomenon is partly explained by elevated vascular adhesion protein-1 and α4β7-integrin in liver tissue of patients with PSC and could lead to a bidirectional inflammatory link between liver and gut [44]. This may partly explain the observed effectiveness of vedolizumab (an α4β7-integrin inhibitor) in PSC-IBD, but studies have not observed an improvement of liver tests in patients with PSC receiving vedolizumab [12, 42]. Another hypothesis is alteration in bile signalling. The primary bile acids, produced by the liver and excreted in the bile, are mostly reabsorbed in the enterohepatic circulation. The remaining bile acids are processed to secondary and tertiary bile acids by the intestinal microbiota [10, 45]. While in IBD alone more primary bile acids are found, patients with PSC-IBD have increased conjugated bile acids like glycochenodeoxycholic acid (GCDCA) [40, 46]. Bile acids signal through specific receptors (e.g. farnesoid X receptor or TGR5) and promote the inflammatory cascade, for example, via NF-κB signalling, which is likely not completely targeted via IL-12/IL-23 blockage, but probably to a higher extent by TNF-α blockers [45]. Together, there are distinctive pathophysiological features of PSC-IBD, which may influence the treatment response to anti-IL-12/-23 agents like ustekinumab or other biological drugs.

Our study provides long-term follow-up data under real-world conditions, directly comparing patients with PSC-IBD and patients with IBD alone. However, several limitations warrant consideration. While the matching process balanced baseline variables overall, some imbalances persisted in the treatment subgroups and residual confounding cannot be fully excluded. The small sample size, particularly in subgroups, and the retrospective study design, including the retrospective scoring of pMS and HBI, should be noted as the main limiting factors. Additionally, colonoscopy or FC assessment was available for only a limited number of patients, which is particularly relevant given the limited correlation between clinical and objective assessment in patients with PSC-IBD. Finally, ustekinumab was mostly given to bio-exposed patients, who may reflect a more treatment-refractory patient population. Nonetheless, the reduced ustekinumab efficacy remained significant in sensitivity analyses.

PSC-IBD is a rare condition, and analyses often rely on relatively small cohorts, which may influence the interpretation of results. The limited sample size of our study may therefore mask certain differences between groups. We hope our findings will encourage future prospective and/or multicentre research to investigate this population, especially the endoscopic and histologic response, in larger cohorts. Beyond clinical trials, further research into the pathophysiology of PSC-IBD would help tailor biological treatments for this challenging patient population.

## Conclusions

Infliximab and vedolizumab are effective in achieving clinical response in patients with PSC-IBD, while ustekinumab is less effective in these patients. However, given the limitations of this study, future prospective and/or multicentre research with larger sample sizes are needed. Meanwhile, further research into the pathophysiology of PSC-IBD will help facilitate precision therapeutic strategies for this challenging patient population.

## Supplementary Material

goag079_Supplementary_Data
