# Supplementary material for: Real-world data on clinical response to inflammatory bowel disease biological treatments in patients with concurrent primary sclerosing cholangitis: a case–control study
Source: Gastroenterol Rep (Oxf). 2026 Jul 30;14:goag079. doi: 10.1093/gastro/goag079 (PMC13423238; doi:10.1093/gastro/goag079)
Supplement: goag079_Supplementary_Data [file goag079_supplementary_data.docx]

**Supplementary Material**

**Supplementary Table 1.** Subgroups of patients with and without liver cirrhosis within the PSC-IBD group.

| **Variables** | PSC-IBD patients with liver cirrhosis (*n* = 15) | PSC-IBD patients without liver cirrhosis (*n* = 31) | *P*-value |
| --- | --- | --- | --- |
| Age, years | 41 (33–51) | 37 (32–42) | - |
| Male | 13 (86.7) | 18 (58.1) | - |
| IBD-subtype |  |  | - |
| Ulcerative colitis | 10 (66.7) | 24 (77.4) |  |
| Crohn´s disease | 5 (33.3) | 7 (22.6) |  |
| Child-Pugh classification |  |  | - |
| A | 8 (53.3) | - |  |
| B | 4 (26.7) | - |  |
| C | 1 (6.7) | - |  |
| NA | 2 (13.3) |  |  |
| Duration of liver cirrhosis, years | 3 (2) | - | - |
| Neoplasia |  |  | - |
| Cholangiocarcinoma | 0 (0) | 2 (6.5) |  |
| Hepatocellular carcinoma | 1 (6.7) | 0 (0) |  |
| Liver transplantation | 7 (46.7) | 4 (12.9) | - |
| Infliximab therapy | 8 | 11 |  |
| Remission | 5 (62.5) | 8 (72.7) | 1.000 |
| Partial response | 1 (12.5) | 1 (9.1) |  |
| No response | 2 (25.0) | 2 (18.2) |  |
| Vedolizumab therapy | 11 | 17 |  |
| Remission | 6 (54.6) | 13 (76.5) | 0.353 |
| Partial response | 2 (18.2) | 2 (11.8) |  |
| No response | 3 (27.3) | 2 (11.8) |  |
| Ustekinumab therapy | 8 | 13 |  |
| Remission | 4 (50.0) | 4 (30.8) | 0.673 |
| Partial response | 0 (0) | 1 (7.7) |  |
| No response | 4 (50.0) | 8 (61.5) |  |
| The data are presented as median (interquartile range) or *n* (percentage). PSC-IBD, primary sclerosing cholangitis associated inflammatory bowel disease; NA, not available. | | | |

**Supplementary Table 2.** Further characteristics of the cohort in this study.

| Variables | Original population | |  | Matched population | |
| --- | --- | --- | --- | --- | --- |
|  | PSC-IBD  (*n* = 46) | IBD  (*n* = 180) |  | PSC-IBD  (*n* = 46) | IBD  (*n* = 46) |
| IBD-data | | | | | |
| Surgical IBD-Therapies |  |  |  |  |  |
| Small bowel resection | 0 (0.0) | 7 (3.9) |  | 0 (0.0) | 2 (4.3) |
| Ileocecal resection | 1 (2.2) | 23 (12.8) |  | 1 (2.2) | 5 (10.9) |
| Colectomy | 4 (8.7) | 5 (2.8) |  | 4 (8.7) | 1 (2.2) |
| Hemicolectomy | 1 (2.2) | 7 (3.9) |  | 1 (2.2) | 1 (2.2) |
| Subtotal colectomy | 2 (4.3) | 3 (1.7) |  | 2 (4.3) | 0 (0.0) |
| Sigmoidectomy | 0 (0.0) | 9 (5.0) |  | 0 (0.0) | 0 (0.0) |
| Rectal resection | 0 (0.0) | 2 (1.1) |  | 0 (0.0) | 0 (0.0) |
| Extraintestinal manifestations |  |  |  |  |  |
| Arthralgia | 7 (15.2) | 33 (18.4) |  | 7 (15.2) | 8 (17.4) |
| Uveitis | 2 (4.3) | 0 (0.0) |  | 2 (4.3) | 0 (0.0) |
| Nephritis | 0 (0.0) | 2 (1.1) |  | 0 (0.0) | 2 (4.3) |
| Pulmonary hypertension | 1 (2.2) | 0 (0.0) |  | 1 (2.2) | 0 (0.0) |
| Cutaneous leukocytoclastic vasculitis | 0 (0.0) | 1 (0.6) |  | 0 (0.0) | 0 (0.0) |
| Erythema nodosum | 4 (8.7) | 4 (2.2) |  | 4 (8.7) | 2 (4.3) |
| Pyoderma gangraenosum | 0 (0.0) | 1 (0.6) |  | 0 (0.0) | 1 (2.2) |
| Recurrent conjunctivitis | 0 (0.0) | 1 (0.6) |  | 0 (0.0) | 1 (2.2) |
| Pharmacological treatment of IBD (without biologicals) |  |  |  |  |  |
| Azathioprine | 28 (60.9) | 101 (56.1) |  | 28 (60.9) | 30 (65.2) |
| Budesonide | 12 (26.1) | 61 (33.9) |  | 12 (26.1) | 14 (30.4) |
| Ciclosporin A | 0 (0.0) | 3 (1.7) |  | 0 (0.0) | 1 (2.2) |
| Immunosuppressants for transplant | 11 (23.9) | 1 (0.6) |  | 11 (23.9) | 0 (0.0) |
| Mercaptopurine | 1 (2.2) | 2 (1.1) |  | 1 (2.2) | 0 (0.0) |
| Mesalazine | 36 (78.3) | 136 (75.6) |  | 36 (78.3) | 35 (76.1) |
| Methotrexate | 3 (6.5) | 7 (3.9) |  | 3 (6.5) | 0 (0.0) |
| Mycophenolate mofetil | 3 (6.5) | 0 (0.0) |  | 3 (6.5) | 0 (0.0) |
| Steroids | 8 (17.4) | 77 (42.8) |  | 8 (17.4) | 20 (43.5) |
| Sulfasalazine | 2 (4.3) | 6 (3.3) |  | 2 (4.3) | 1 (2.2) |
| Tacrolimus | 2 (4.3) | 6 (3.3) |  | 2 (4.3) | 2 (4.3) |
| Complications of IBD |  |  |  |  |  |
| Colorectal cancer | 1 (2.2) | 0 (0.0) |  | 1 (2.2) | 0 (0.0) |
| No complications | 45 (97.8) | 180 (100.0) |  | 45 (97.8) | 46 (100.0) |
| PSC-data | | | | | |
| Age at initial diagnosis, years | 25 (19–32) | - |  | 25 (19–32) | - |
| Duration of PSC, years | 11 (6–18) | - |  | 11 (6–18) | - |
| Overlap |  |  |  |  |  |
| Autoimmune hepatitis | 9 (19.6) | - |  | 9 (19.6) | - |
| Primary biliary cholangitis | 1 (2.2) | - |  | 1 (2.2) | - |
| No overlap | 36 (78.3) | - |  | 36 (78.3) | - |
| Complications of PSC |  |  |  |  |  |
| Cholangiocarcinoma | 2 (4.3) | - |  | 2 (4.3) | - |
| Hepatocellular carcinoma | 1 (2.2) | - |  | 1 (2.2) | - |
| Pancreatitis | 3 (6.5) | - |  | 3 (6.5) | - |
| Hepatic cirrhosis | 15 (32.6) | - |  | 15 (32.6) | - |
| No complications | 28 (60.9) | - |  | 28 (60.9) | - |
| Age at initial diagnosis of cirrhosis, years | 22 (22–61) | - |  | 22 (22–61) | - |
| Duration of cirrhosis, years | 3 (2–4) | - |  | 3 (2–4) | - |
| Child-Pugh-Score |  |  |  |  |  |
| Child A | 8 (17.4) | - |  | 8 (17.4) | - |
| Child B | 4 (8.7) | - |  | 4 (8.7) | - |
| Child C  NA | 1 (2.2)  2 (4.3) | - |  | 1 (2.2)  2 (4.3) | - |
| Hepatic transplantation | 11 (23.9) | - |  | 11 (23.9) | - |
| Complications of hepatic transplantation |  |  |  |  |  |
| Ischemic type biliary lesions | 1 (2.2) | - |  | 1 (2.2) | - |
| Mild cellular rejection | 1 (2.2) | - |  | 1 (2.2) | - |
| PSC relapse | 1 (2.2) | - |  | 1 (2.2) | - |
| No complications | 8 (17.4) | - |  | 8 (17.4) | - |

The data are presented as median (interquartile range) or *n* (percentage). IBD, Inflammatory bowel disease; PSC, primary sclerosing cholangitis; NA, not available.

**Supplementary Table 3.** Cohort demographics and disease characteristics among each treatment subgroup.

| Variables | Matched patients treated with infliximab | | |  | Matched patients treated with vedolizumab | | |  | Matched patients treated with ustekinumab | | |
| --- | --- | --- | --- | --- | --- | --- | --- | --- | --- | --- | --- |
|  | PSC-IBD  (*n* = 19) | IBD  (*n* = 20) | SMD |  | PSC-IBD  (*n* = 28) | IBD  (*n* = 22) | SMD |  | PSC-IBD  (*n* = 21) | IBD  (*n* = 26) | SMD |
| Age, years | 33 (26–42) | 36.5 (26–44.5) | 0.23 |  | 38 (33.5–49) | 32 (24–42) | 0.48 |  | 38 (32–44) | 38 (27–48) | 0.08 |
| Female | 8 (42.1) | 12 (60.0) | 0.36 |  | 7 (25.0) | 10 (45.5) | 0.43 |  | 6 (28.6) | 17 (65.4) | 0.8 |
| Body mass index, kg/m^2^ | 21.7 (20.2–24.1) | 23.3 (20.7–26.6) | 0.48 |  | 22.4 (20.3–25.0) | 22.3 (18.8–26.9) | 0.08 |  | 22.2 (20.9–23.4) | 22.2 (19.2–26.2) | 0.04 |
| Smoker |  |  | 0.27 |  |  |  | 0.34 |  |  |  | 0.52 |
| Yes | 5 (26.3) | 7 (38.9) |  |  | 3 (11.1) | 5 (23.8) |  |  | 4 (20.0) | 10 (43.5) |  |
| No | 14 (73.7) | 11 (61.1) |  |  | 24 (88.9) | 16 (76.2) |  |  | 16 (80.0) | 13 (56.5) |  |
| CCI, mean ± standard deviation | 0.4 ± 0.8 | 0.3 ± 0.6 | 0.17 |  | 0.6 ± 1.3 | 0.4 ± 1.0 | 0.2 |  | 0.6 ± 0.9 | 0.7 ± 1.2 | 0.15 |
| IBD subtype |  |  | 0.49 |  |  |  | 0.16 |  |  |  | 0.11 |
| UC | 11 (57.9) | 16 (80.0) |  |  | 24 (85.7) | 20 (90.9) |  |  | 14 (66.7) | 16 (61.5) |  |
| CD | 8 (42.1) | 4 (20.0) |  |  | 4 (14.3) | 2 (9.1) |  |  | 7 (33.3) | 10 (38.5) |  |
| Age at initial diagnosis, years | 24 (16–30) | 19 (15.5–31.5) | 0.14 |  | 23 (17.5–26.5) | 20.5 (18–25) | 0.04 |  | 23.0 (18–27) | 21.5 (18–28) | 0.04 |
| Duration of IBD, years | 11 (6–13) | 11 (5–23.5) | 0.42 |  | 16 (11.5–24) | 8.5 (5–15) | 0.57 |  | 13.0 (11–18) | 11.0 (5–20) | 0.06 |
| Montreal Classification of UC |  |  | 0.53* |  |  |  | 0.45* |  |  |  | 0.82* |
| Proctitis | 0 (0.0) | 0 (0.0) |  |  | 1 (4.3) | 2 (10.0) |  |  | 0 (0.0) | 0 (0.0) |  |
| Left-sided | 0 (0.0) | 2 (12.5) |  |  | 1 (4.3) | 3 (15.0) |  |  | 0 (0.0) | 4 (25.0) |  |
| Pancolitis | 11 (100.0) | 14 (87.5) |  |  | 21 (91.3) | 15 (75.0) |  |  | 14 (100.0) | 12 (75.0) |  |
| Montreal Classification of CD |  |  | 0.00** |  |  |  | 1.00** |  |  |  | 0.06** |
| Ileal | 1 (12.5) | 1 (25.0) |  |  | 0 | 1 (50.0) |  |  | 1 (14.3) | 3 (30.0) |  |
| Colon | 3 (37.5) | 1 (25.0) |  |  | 2 (66.6) | 1 (50.0) |  |  | 2 (28.6) | 1 (10.0) |  |
| Ileocolon | 4 (50.0) | 2 (50.0) |  |  | 1 (33.3) | 0 (0.0) |  |  | 4 (57.1) | 6 (60.0) |  |

The data are presented as median (interquartile range) or *n* (percentage), except CCI. CCI, Charlson Comorbidity Index (Modification Quan *et al.*). PSC, primary sclerosing cholangitis; IBD, inflammatory bowel disease; UC, ulcerative colitis; CD, Crohn’s disease; SMD, standardized mean difference; SMD < 0.1 indicates well balanced, < 0.2 indicates balanced, > 0.2 indicates potential bias; *, SMD calculated pancolitis vs no pancolitis; **, SMD calculated ileocolon vs no ileocolon.

**Supplementary Table 4.** Characteristics of each treatment group.

| Variables | Infliximab | | |  | Vedolizumab | | |  | Ustekinumab | | |
| --- | --- | --- | --- | --- | --- | --- | --- | --- | --- | --- | --- |
|  | PSC-IBD  (*n* = 19) | IBD  (*n* = 20) | *P*-value |  | PSC-IBD  (*n* = 28) | IBD  (*n* = 22) | *P*-value |  | PSC-IBD  (*n* = 21) | IBD  (*n* = 26) | *P*-value |
| Reasons for ending biological therapy |  |  | - |  |  |  | - |  |  |  | - |
| Non-responder |  |  |  |  |  |  |  |  |  |  |  |
| Primary non-responder | 4 (21.1) | 5 (25.0) |  |  | 5 (17.9) | 3 (13.6) |  |  | 12 (57.1) | 6 (23.1) |  |
| Loss of response | 7 (36.8) | 6 (30.0) |  |  | 13 (46.4) | 14 (63.6) |  |  | 2 (9.5) | 7 (26.9) |  |
| Other disease | 0 (0) | 0 (0) |  |  | 0 (0) | 1 (4.6) |  |  | 0 (0) | 0 (0) |  |
| Hepatic transplantation | 0 (0) | 0 (0) |  |  | 1 (3.6) | 0 (0.0) |  |  | 0 (0) | 0 (0) |  |
| Adverse drug reaction | 2 (10.5) | 5 (25.0) |  |  | 1 (3.6) | 0 (0.0) |  |  | 1 (4.8) | 1 (3.9) |  |
| Infect complications | 1 | 0 |  |  | 1 | 0 |  |  | 0 | 1 |  |
| Lupus like Syndrome | 0 | 1 |  |  | 0 | 0 |  |  | 0 | 0 |  |
| Skin complications | 0 | 3 |  |  | 0 | 0 |  |  | 1 | 0 |  |
| Tingling sensation | 0 | 1 |  |  | 0 | 0 |  |  | 0 | 0 |  |
| Pruritus | 1 | 0 |  |  | 0 | 0 |  |  | 0 | 0 |  |
| Request of the patient | 1 (5.3) | 0 (0) |  |  | 1 (3.6) | 0 (0.0) |  |  | 2 (9.5) | 0 (0.0) |  |
| Patient died | 0 (0) | 0 (0) |  |  | 3 (10.7) | 0 (0.0) |  |  | 0 (0.0) | 0 (0.0) |  |
| Ongoing therapy^a^ | 5 (26.3) | 4 (20.0) |  |  | 4 (14.3) | 4 (18.2) |  |  | 4 (19.0) | 12 (46.2) |  |
| Baseline partial Mayo Score, median (IQR) | 5 (3–6) | 5 (3–7) | - |  | 3 (1–6) | 3 (2–5) | - |  | 4 (3–6) | 4 (2–7) | - |
| Missing number in baseline partial Mayo Score | 0 | 2 | - |  | 8 | 3 | - |  | 3 | 1 | - |
| Baseline Harvey Bradshaw Index, median (IQR) | 2 (2–3) | 10 (5.5–18) | - |  | 11 (11–11) | 8 (8–8) | - |  | 7 (4–7) | 5 (1–11) | - |
| Missing number in baseline Harvey Bradshaw Index | 5 | 0 | - |  | 3 | 1 | - |  | 2 | 1 | - |
| Line of therapy |  |  | 0.425 (Fisher´s exact test) |  |  |  | 0.636 (Fisher´s exact test) |  |  |  | 0.164 (Fisher´s exact test) |
| First-line | 12 (63.2) | 13 (65.0) |  |  | 16 (57.1) | 14 (63.6) |  |  | 2 (9.5) | 9 (34.6) |  |
| Second-line | 2 (10.5) | 5 (25.0) |  |  | 9 (32.1) | 6 (27.3) |  |  | 9 (42.9) | 6 (23.1) |  |
| Third-line | 4 (21.1) | 2 (10.0) |  |  | 3 (10.7) | 1 (4.6) |  |  | 5 (23.8) | 8 (30.8) |  |
| Fourth-line | 1 (5.3) | 0 (0) |  |  | 0 (0) | 1 (4.6) |  |  | 4 (19.0) | 2 (7.7) |  |
| Fifth-line | 0 (0) | 0 (0) |  |  | 0 (0) | 0 (0) |  |  | 1 (4.8) | 1 (3.9) |  |
| Steroid use within the first 20 weeks of therapy |  |  |  |  |  |  |  |  |  |  |  |
| Yes | 9 (52.9) | 11 (57.9) | 0.765 |  | 12 (54.5) | 10 (47.6) | 0.650 |  | 11 (55.0) | 15 (57.7) | 0.855 |
| No | 8 (47.1) | 8 (42.1) |  |  | 10 (45.5) | 11 (52.4) |  |  | 9 (45.0) | 11 (42.3) |  |
| Missing | 2 | 1 |  |  | 6 | 1 |  |  | 1 | 0 |  |
| Concomitant use of other IBD-therapy medication over the whole therapy period |  |  | 0.341 |  |  |  | 0.253 |  |  |  | 0.052 |
| No concomitant medication | 9 (47.4) | 13 (65.0) |  |  | 10 (35.7) | 12 (54.6) |  |  | 12 (57.1) | 22 (84.6) |  |
| Azathioprine | 4 (21.1) | 5 (25.0) |  |  | 4 (14.3) | 1 (4.6) |  |  | 0 (0.0) | 0 (0.0) |  |
| Budesonide | 2 (10.5) | 1 (5.0) |  |  | 1 (3.6) | 1 (4.6) |  |  | 0 (0.0) | 1 (3.9) |  |
| Immunosuppressants for transplant | 1 (5.3) | 0 (0) |  |  | 3 (10.7) | 0 (0.0) |  |  | 1 (4.8) | 0 (0.0) |  |
| Mesalamine | 7 (36.8) | 3 (15.0) |  |  | 14 (50.0) | 9 (40.9) |  |  | 7 (33.3) | 3 (11.5) |  |
| Mycophenolate mofetil | 0 (0) | 0 (0) |  |  | 2 (7.1) | 0 (0.0) |  |  | 0 (0.0) | 0 (0.0) |  |
| Sulfasalazine | 0 (0) | 0 (0) |  |  | 0 (0.0) | 0 (0.0) |  |  | 1 (4.8) | 0 (0.0) |  |

^a^ Patients continued therapy beyond November 2023 or end of therapy is not known (e.g. patient left city).

Data are presented as median (interquartile range) or number (percentage). PSC, primary sclerosing cholangitis; IBD, inflammatory bowel disease; IQR, interquartile range.

**Supplementary Table 5.** Primary and secondary outcomes.

| Variables | Infliximab | | |  | Vedolizumab | | |  | Ustekinumab | | | | | |  |
| --- | --- | --- | --- | --- | --- | --- | --- | --- | --- | --- | --- | --- | --- | --- | --- |
|  | PSC-IBD  (*n* = 19) | IBD  (*n* = 20) | *P*-value |  | PSC-IBD  (*n* = 28) | IBD  (*n* = 22) | *P*-value |  | PSC-IBD  (*n* = 21) | | IBD  (*n* = 26) | | *P*-value | |  |
| IBD-subtype |  |  |  |  |  |  |  |  | |  | |  | |  | |
| UC | 11 (57.9) | 16 (80.0) |  |  | 24 (85.7) | 20 (90.9) |  |  | | 14 (66.7) | | 16 (61.5) | |  | |
| CD | 8 (42.1) | 4 (20.0) |  |  | 4 (14.3) | 2 (9.1) |  |  | | 7 (33.3) | | 10 (38.5) | |  | |
| Primary outcome: Clinical response to biological therapy within the first 20 weeks^a^ | | | | | | | | | | | | | | | |
| Remission | 13 (68.4) | 14 (70.0) | 1.000 (Fisher´s exact test) |  | 19 (67.9) | 18 (81.8) | 1.000 (Fisher´s Exact Test) |  | 8 (38.1) | | 18 (69.2) | | 0.017  (Chi-square test) | |  |
| Partial response | 2 (10.5) | 1 (5.0) |  |  | 4 (14.3) | 1 (4.6) |  |  | 1 (4.8) | | 2 (7.7) | |  |  |  |
| Primary non-response | 4 (21.1) | 5 (25.0) |  |  | 5 (17.9) | 3 (13.6) |  |  | 12 (57.1) | | 6 (23.1) | |  |  |  |
| Secondary outcomes: Clinical response in the course, endoscopic response and laboratory values | | | | | | | | | | | | | | |  |
| Therapy status after 12 months |  |  | 0.758  (therapy ongoing vs rest) |  |  |  | 0.981 (therapy ongoing vs rest) |  |  | |  | | 0.252 (therapy ongoing vs rest) | |  |
| Remission | 9 (50.0) | 11 (55.0) |  |  | 17 (60.7) | 14 (63.6) |  |  | 6 (28.6) | | 10 (45.5) | |  | |  |
| Partial response | 0 (0) | 0 (0) |  |  | 2 (7.1) | 1 (4.6) |  |  | 0 (0) | | 0 (0) | |  | |  |
| Primary non-response | 4 (22.2) | 5 (25.0) |  |  | 5 (17.9) | 3 (13.6) |  |  | 12 (57.1) | | 6 (27.3) | |  | |  |
| Loss of response | 5 (27.8) | 3 (15.0) |  |  | 3 (10.7) | 3 (13.6) |  |  | 2 (9.5) | | 6 (27.3) | |  | |  |
| Therapy discontinuation due to other reason | 0 (0) | 1 (5.0) |  |  | 1 (3.6) | 1 (4.6) |  |  | 1 (4.8) | | 0 (0) | |  | |  |
| Missing (because of too short follow-up) | 1 | 0 |  |  | 0 | 0 |  |  | 0 | | 4 | |  | |  |
| Overall duration^b^, mean ± SD, months |  |  |  |  |  |  |  |  |  | |  | |  | |  |
| All patients | 14 ± 11 | 18 ± 22 |  |  | 20 ± 17 | 26 ± 22 |  |  | 7 ± 4 | | 10 ± 10 | |  | |  |
| Patients with primary non-response | 7 ± 3 | 3 ± 2 |  |  | 4 ± 1 | 4 ± 1 |  |  | 4 ± 2 | | 3 ± 2 | |  | |  |
| Patients with loss of response | 13 ± 11 | 16 ± 9 |  |  | 25 ± 18 | 32 ± 21 |  |  | 13 ± 3 | | 12 ± 9 | |  | |  |
| Therapy end due to other reasons | 26 ± 12 | 36 ± 31 |  |  | 24 ± 15 | 8 ± 0 |  |  | | 11 ± 4 | | 32 ± 0 | |  | |
| Duration until now^c^, mean ± SD, months | 36 ± 37 | 61 ± 80 |  |  | 64 ± 35 | 64 ± 37 |  |  | | 18 ± 10 | | 25 ± 21 | |  | |
| Endoscopic response^d^ |  |  |  |  |  |  |  |  | |  | |  | |  | |
| Mucosal healing during the first 12 months | 2 (33.3) | 2 (28.6) | 1.000 |  | 6 (35.3) | 5 (55.6) | 0.419 |  | 1 (11.1) | | 2 (28.6) | | 0.550 | |  |
| No colonoscopy during the first 12 months | 13 | 13 |  |  | 11 | 13 |  |  | 12 | | 19 | |  | |  |
| Mucosal healing at all | 4 (40.0) | 7 (58.3) | 0.670 |  | 9 (39.1) | 11 (57.9) | 0.352 |  | 2 (20.0) | | 10 (66.7) | | 0.041 | |  |
| No colonoscopy at all | 9 | 8 |  |  | 5 | 3 |  |  | 11 | | 11 | |  | |  |
| Therapy response based on faecal calprotectin^e^ |  |  |  |  |  |  |  |  |  | |  | |  | |  |
| Calprotectin-response during the first 12 months | 6 (75.0) | 5 (62.5) | 1.000 |  | 4 (50.0) | 5 (55.6) | 1.000 |  | 2 (20.0) | | 7 (50.0) | | 0.210 | |  |
| Calprotectin not available during the first 12 months | 11 | 12 |  |  | 20 | 13 |  |  | 11 | | 12 | |  | |  |
| Calprotectin-response at all | 6 (75.0) | 6 (60.0) | 0.638 |  | 7 (63.6) | 8 (66.6) | 1.000 |  | 2 (20.0) | | 9 (56.3) | | 0.109 | |  |
| No calprotectin values | 11 | 10 |  |  | 17 | 10 |  |  | 11 | | 10 | |  | |  |

Data are presented as number (percentage), except duration. IBD, inflammatory bowel disease; UC, ulcerative colitis; CD, Crohn’s disease; SD, standard deviation.

^a^Clinical response was evaluated using partial Mayo Score (pMS) for UC and Harvey Bradshaw-Index (HBI) for CD: remission: pMS ≤ 1/HBI < 5; particular response: a decrease of pMS ≥ 2 or a decrease of HBI > 3; no-response.

^b^ Patients finished therapy until November 2023.

^c^ Patients continued therapy beyond November 2023.

^d^ Endoscopic response was evaluated using colonoscopy and histology findings: mucosal healing: no or mild inflammation.

^e^ This was evaluated using faecal calprotectin (FC) values: response: FC < 150 µg/g or decreased by more than 50% compared with baseline.

**Supplementary Table 6.** Characteristics of each treatment group (patients with UC only).

| Variables | Infliximab | | |  | Vedolizumab | | |  | Ustekinumab | | |  |
| --- | --- | --- | --- | --- | --- | --- | --- | --- | --- | --- | --- | --- |
|  | PSC-UC  (*n* = 11) | UC  (*n* = 16) | *P*-value |  | PSC-UC  (*n* = 24) | UC  (*n* = 20) | *P*-value |  | PSC-UC  (*n* = 14) | UC  (*n* = 16) | *P*-value |  |
| Reasons ending biological therapy |  |  |  |  |  |  |  |  |  |  |  |  |
| Non-responder |  |  |  |  |  |  |  |  |  |  |  |  |
| Primary non-responder | 2 (18.2) | 4 (25.0) |  |  | 4 (16.7) | 2 (10.0) |  |  | 9 (64.3) | 5 (31.3) |  |  |
| Loss of response | 5 (45.5) | 4 (25.0) |  |  | 12 (50.0) | 13 (65.0) |  |  | 1 (7.1) | 3 (18.8) |  |  |
| Other disease | 0 (0.0) | 0 (0.0) |  |  | 0 (0.0) | 1 (5.0) |  |  | 0 (0.0) | 0 (0.0) |  |  |
| Hepatic transplantation | 0 (0.0) | 0 (0.0) |  |  | 0 (0.0) | 0 (0.0) |  |  | 0 (0.0) | 0 (0.0) |  |  |
| Adverse drug reaction |  |  |  |  |  |  |  |  |  |  |  |  |
| All adverse reactions | 0 (0.0) | 4 (25.0) |  |  | 1 (4.2) | 0 (0.0) |  |  | 0 (0.0) | 0 (0.0) |  |  |
| Infection complications | 0 | 0 |  |  | 1 | 0 |  |  | 0 | 0 |  |  |
| Skin complications | 0 | 3 |  |  | 0 | 0 |  |  | 0 | 0 |  |  |
| Tingling sensation | 0 | 1 |  |  | 0 | 0 |  |  | 0 | 0 |  |  |
| Request of the patient | 1 (9.1) | 0 (0.0) |  |  | 1 (4.2) | 0 (0.0) |  |  | 1 (7.1) | 0 (0.0) |  |  |
| Patient dead | 0 (0.0) | 0 (0.0) |  |  | 3 (12.5) | 0 (0.0) |  |  | 0 (0.0) | 0 (0.0) |  |  |
| Ongoing therapy^a^ | 3 (27.3) | 4 (25.0) |  |  | 3 (12.5) | 4 (20.0) |  |  | 3 (21.4) | 8 (50.0) |  |  |
| Baseline partial Mayo Score |  |  |  |  |  |  |  |  |  |  |  |  |
| Median | 5 (3–6) | 5 (3–7) |  |  | 3 (1–6) | 3 (2–5) |  |  | 4 (3–6) | 4 (2–7) |  |  |
| Missing | 0 | 2 |  |  | 8 | 3 |  |  | 3 | 1 |  | |
| Line of therapy |  |  | 0.502 (Fisher´s exact test) |  |  |  | 0.357 (Fisher´s exact test) |  |  |  | 0.052 (Fisher´s exact test) | |
| First-line | 6 (54.6) | 11 (68.8) |  |  | 14 (58.3) | 13 (65.0) |  |  | 0 (0.0) | 3 (18.8) |  | |
| Second-line | 1 (9.1) | 3 (18.8) |  |  | 7 (29.2) | 6 (30.0) |  |  | 6 (42.9) | 5 (31.3) |  | |
| Third-line | 3 (27.3) | 2 (12.5) |  |  | 3 (12.5) | 0 (0.0) |  |  | 3 (21.4) | 7 (43.8) |  | |
| Fourth-line | 1 (9.1) | 0 (0.0) |  |  | 0 (0.0) | 1 (5.0) |  |  | 4 (28.6) | 0 (0.0) |  | |
| Fifth-line | 0 (0.0) | 0 (0.0) |  |  | 0 (0.0) | 0 (0.0) |  |  | 1 (7.1) | 1 (6.3) |  | |
| Steroid use within the first 20 weeks of therapy |  |  | 1.000 |  |  |  | 0.621 |  |  |  | 1.000 | |
| Yes | 7 (63.6) | 10 (66.6) |  |  | 10 (50.0) | 8 (42.1) |  |  | 9 (69.2) | 10 (62.5) |  | |
| No | 4 (36.4) | 5 (33.3) |  |  | 10 (50.0) | 11 (57.9) |  |  | 4 (30.8) | 6 (37.5) |  | |
| Missing | 0 | 1 |  |  | 4 | 1 |  |  | 1 | 0 |  | |
| Concomitant use of other IBD-therapy medication over the whole therapy |  |  | 0.130 |  |  |  | 0.125 |  |  |  | 0.135 | |
| No concomitant medication | 4 (36.4) | 11 (68.8) |  |  | 7 (29.17) | 11 (55.0) |  |  | 6 (42.9) | 12 (75.0) |  | |
| Azathioprine | 2 (18.2) | 4 (25.0) |  |  | 3 (12.5) | 1 (5.0) |  |  | 0 (0.0) | 0 (0.0) |  | |
| Budesonide | 2 (18.2) | 0 (0.0) |  |  | 1 (4.2) | 1 (5.0) |  |  | 0 (0.0) | 1 (6.3) |  | |
| Immunosuppressants for transplant | 1 (9.1) | 0 (0.0) |  |  | 3 (12.5) | 0 (0.0) |  |  | 1 (7.1) | 0 (0.0) |  | |
| Mesalamine | 5 (45.5) | 3 (18.8) |  |  | 13 (54.2) | 8 (40.0) |  |  | 7 (50.0) | 3 (18.8) |  | |
| Mycophenolate mofetil | 0 (0.0) | 0 (0.0) |  |  | 1 (4.2) | 0 (0.0) |  |  | 0 (0.0) | 0 (0.0) |  | |
| Sulfasalazine | 0 (0.0) | 0 (0.0) |  |  | 0 (0.0) | 0 (0.0) |  |  | 0 (0.0) | - |  | |

Data are presented as median (interquartile range) or number (percentage). PSC, primary sclerosing cholangitis; IBD, inflammatory bowel disease; UC, ulcerative colitis.

^a^ Patients continued therapy beyond November 2023 or end of therapy is not known (e.g. patient left city).

**Supplementary Table 7.** Primary and secondary outcomes (patients with UC only).

| Variables | Infliximab | | |  | Vedolizumab | | | | |  | Ustekinumab | | |
| --- | --- | --- | --- | --- | --- | --- | --- | --- | --- | --- | --- | --- | --- |
|  | PSC-UC  (*n* = 11) | UC  (*n* = 16) | *P*-value |  | PSC-UC  (*n* = 24) | | UC  (*n* = 20) | | *P*-value |  | PSC-UC  (*n* = 14) | UC  (*n* = 16) | *P*-value |
| Primary outcome: clinical response to biological therapy within the first 20 weeks^a^ | | | | | | | | | | | | | |
| Clinical response |  |  | 1.000 (Fisher´s exact test) |  |  | |  | | 0.673 (Fisher´s exact test) |  |  |  | 0.070  (Chi-square test) |
| Remission | 7 (63.6) | 12 (75.0) |  |  | 17 (70.8) | | 17 (85.0) | |  |  | 4 (28.6) | 10 (62.5) |  |
| Partial response | 2 (18.2) | 0 (0) |  |  | 3 (12.5) | | 1 (5.0) | |  |  | 1 (7.1) | 1 (6.3) |  |
| Primary non-response | 2 (18.2) | 4 (25.0) |  |  | 4 (16.7) | | 2 (10.0) | |  |  | 9 (64.3) | 5 (31.3) |  |
| Secondary outcomes: clinical response in the course, endoscopic response and laboratory values | | | | | | | | | | | | | |
| Therapy status after 12 months |  |  | 0.428  (Fisher´s exact test) |  |  |  | | 1.000 (Fisher´s exact test) | |  |  |  | 0.665 (Fisher´s exact test) |
| Remission | 3 (30.0) | 8 (50.0) |  |  | 16 (66.7) | 14 (70.0) | |  | |  | 3 (21.4) | 4 (33.3) |  |
| Partial response | 0 (0) | 0 (0) |  |  | 1 (4.2) | 1 (5.0) | |  | |  | 0 (0) | 0 (0) |  |
| Primary non-response | 2 (20.0) | 4 (25.0) |  |  | 4 (16.7) | 2 (10.0) | |  | |  | 9 (64.3) | 5 (41.7) |  |
| Loss of response | 5 (50.0) | 3 (18.8) |  |  | 2 (8.3) | 2 (10.0) | |  | |  | 1 (7.1) | 3 (25.0) |  |
| Therapy discontinuation due to other reason | 0 | 1 (6.3) |  |  | 1 (4.2) | 1 (5.0) | |  | |  | 1 (7.1) | 0 (0.0) |  |
| Missing (because of too short follow-up) | 1 | 0 |  |  | 0 | 0 | |  | |  | 0 | 4 |  |
| Overall duration^b^, mean ± SD, months |  |  |  |  |  |  | |  | |  |  |  |  |
| All patients | 11 ± 11 | 17± 24 |  |  | 21 ± 16 | 29 ± 22 | |  | |  | 5 ± 3 | 6 ± 4 |  |
| Patients with primary non-response | 9 ± 2 | 2 ± 1 |  |  | 4 ± 1 | 4 ± 1 | |  | |  | 5 ± 2 | 4 ± 2 |  |
| Patients with loss of response | 7 ± 4 | 12 ± 8 |  |  | 27 ± 18 | 34 ± 21 | |  | |  | 11 ± 0 | 9 ± 4 |  |
| Therapy end due to other reasons | 36 ± 0 | 36 ± 36 |  |  | 19 ± 9 | 8 ± 0 | |  | |  | 6 ± 0 | - |  |
| Duration until now^c^, mean ± SD, months | 21 ± 19 | 61 ± 80 |  |  | 81 ± 12 | 64 ± 37 | |  | |  | 18 ± 12 | 18 ± 16 |  |
| Endoscopic response^d^ |  |  |  |  |  |  | |  | |  |  |  |  |
| Mucosal healing during the first 12 months | 2 (40.0) | 0 (0) | 0.444 |  | 6 (37.5) | 5 (71.4) | | 0.193 | |  | 1 (14.3) | 1 (25.0) | 1.000 |
| Colonoscopy not available during the first 12 months | 6 | 11 |  |  | 8 | 13 | |  | |  | 7 | 12 |  |
| Mucosal healing at all | 3 (50.0) | 4 (44.4) | 1.000 |  | 8 (38.1) | 11 (64.7) | | 0.191 | |  | 1 (14.3) | 5 (62.5) | 0.119 |
| No colonoscopy at all | 5 | 7 |  |  | 3 | 3 | |  | |  | 7 | 8 |  |
| Therapy response based on faecal calprotectin^e^ |  |  |  |  |  |  | |  | |  |  |  |  |
| Calprotectin-response during the first 12 months | 3 (60.0) | 5 (62.5) | 1.000 |  | 4 (50.0) | 5 (55.6) | | 1.000 | |  | 0 (0.0) | 3 (33.3) | 0.213 |
| No calprotectin values during the first 12 months | 6 | 8 |  |  | 16 | 11 | |  | |  | 7 | 7 |  |
| Calprotectin-response at all | 3 (60.0) | 6 (66.7) | 1.000 |  | 7 (63.6) | 8 (66.7) | | 1.000 | |  | 0 (0.0) | 4 (40.0) | 0.103 |
| No calprotectin values | 6 | 7 |  |  | 13 | 8 | |  | |  | 7 | 6 |  |

Data are presented as number (percentage), except duration; PSC, primary sclerosing cholangitis; UC, ulcerative colitis; SD, standard deviation.

^a^ Clinical response was evaluated using partial Mayo Score (pMS) for UC: remission: pMS ≤ 1/ HBI < 5; particular response: decrease pMS ≥ 2/ HBI > 3; no-response.

^b^ Patients finished therapy until November 2023.

^c^ Patients continued therapy beyond November 2023.

^d^ Endoscopic response was evaluated using colonoscopy and histology findings: mucosal healing: no or mild inflammation.

^e^ This was evaluated using faecal calprotectin (FC) values: response: FC < 150 µg/g or decreased by more than 50% compared with baseline.

**Supplementary Table 8.** Subgroups of patients based on therapy lines.

| Variables | Infliximab | | | |  | Vedolizumab | | |  | Ustekinumab | | |
| --- | --- | --- | --- | --- | --- | --- | --- | --- | --- | --- | --- | --- |
|  | PSC-IBD | IBD | *P*-value | |  | PSC-IBD | IBD | *P*-value |  | PSC-IBD | IBD | *P*-value |
| All patients (bio-naïve and bio-exposed) |  |  | 1.000 (Fisher´s exact test) | |  |  |  | 1.000 (Fisher´s exact test |  |  |  | 0.017  (Chi-square test) |
| Remission | 13 (68.4) | 14 (70.0) |  | |  | 19 (67.9) | 18 (81.8) |  |  | 8 (38.1) | 18 (69.2) |  |
| Partial response | 2 (10.5) | 1 (5.0) |  | |  | 4 (14.3) | 1 (4.6) |  |  | 1 (4.8) | 2 (7.7) |  |
| No response | 4 (21.1) | 5 (25.0) |  | |  | 5 (17.9) | 3 (13.6) |  |  | 12 (57.1) | 6 (23.1) |  |
| Bio-naïve patients (therapy line 1) |  |  | 0.645 | |  |  |  | 1.000 |  |  |  | 0.182 |
| Remission | 10 (83.3) | 9 (69.2) |  | |  | 11 (68.8) | 11 (78.6) |  |  | 1 (50.0) | 8 (88.9) |  |
| Partial response | 0 (0) | 0 (0) |  | |  | 2 (12.5) | 1 (7.1) |  |  | 0 (0) | 1 (11.1) |  |
| No response | 2 (16.7) | 4 (30.8) |  | |  | 3 (18.8) | 2 (14.3) |  |  | 1 (50.0) | 0 (0) |  |
| Bio-exposed patients (therapy lines 2–5) |  |  | 1.000 | |  |  |  | 1.000 |  |  |  | 0.175 |
| Remission | 3 (42.9) | 5 (71.4) |  | |  | 8 (66.7) | 7 (87.5) |  |  | 7 (36.9) | 10 (58.9) |  |
| Partial response | 2 (28.6) | 1 (14.3) |  | |  | 2 (16.7) | 0 (0) |  |  | 1 (5.3) | 1 (5.9) |  |
| No response | 2 (28.6) | 1 (14.3) |  | |  | 2 (16.7) | 1 (12.5) |  |  | 11 (57.9) | 6 (35.3) |  |
| Therapy lines 1–3 |  |  | 1.000 (Fisher´s exact test) | |  |  |  | 1.000 (Fisher´s exact test) |  |  |  | 0.027 |
| Remission | 12 (66.7) | 14 (70.0) |  |  | | 19 (67.9) | 17 (81.0) |  |  | 6 (37.5) | 17 (73.9) |  |
| Partial response | 2 (11.1) | 1 (5.0) |  |  | | 4 (14.3) | 1 (4.8) |  |  | 1 (6.3) | 1 (4.3) |  |
| No response | 4 (22.2) | 5 (25.0) |  |  | | 5 (17.9) | 3 (14.3) |  |  | 9 (56.3) | 5 (21.7) |  |
| Therapy line |  |  |  |  | |  |  |  |  |  |  |  |
| 1 |  |  | 0.645 |  | |  |  | 1.000 |  |  |  | 0.182 |
| Remission | 10 (83.3) | 9 (69.2) |  |  | | 11 (68.8) | 11 (78.6) |  |  | 1 (50.0) | 8 (88.9) |  |
| Partial response | 0 (0) | 0 (0) |  |  | | 2 (12.5) | 1 (7.1) |  |  | 0 (0) | 1 (11.1) |  |
| No response | 2 (16.7) | 4 (30.8) |  |  | | 3 (18.8) | 2 (14.3) |  |  | 1 (50.0) | 0 (0) |  |
| 2 |  |  | 1.000 |  | |  |  | 1.000 |  |  |  | 0.119 |
| Remission | 1 (50.0) | 3 (60.0) |  |  | | 5 (55.6) | 5 (83.3) |  |  | 2 (22.2) | 5 (83.3) |  |
| Partial response | 0 (0) | 1 (20.0) |  |  | | 2 (22.2) | 0 (0) |  |  | 1 (11.1) | 0 (0) |  |
| No response | 1 (50.0) | 1 (20.0) |  |  | | 2 (22.2) | 1 (16.7) |  |  | 6 (66.7) | 1 (16.7) |  |
| 3 |  |  | 1.000 |  | |  |  | 1.000 |  |  |  | 1.000 |
| Remission | 1 (25.0) | 2 (100.0) |  |  | | 3 (100.0) | 1 (100.0) |  |  | 3 (60.0) | 4 (50.0) |  |
| Partial response | 2 (50.0) | 0 (0) |  |  | | 0 (0) | 0 (0) |  |  | 0 (0) | 0 (0) |  |
| No response | 1 (25.0) | 0 (0) |  |  | | 0 (0) | 0 (0) |  |  | 2 (40.0) | 4 (50.0) |  |
| 4 |  |  | - |  | |  |  | - |  |  |  | 0.467 |
| Remission | 1 (100.0) | 0 (0) |  |  | | 0 (0) | 1 (100.0) |  |  | 2 (50.0) | 1 (50.0) |  |
| Partial response | 0 (0) | 0 (0) |  |  | | 0 (0) | 0 (0) |  |  | 0 (0) | 1 (50.0) |  |
| No response | 0 (0) | 0 (0) |  |  | | 0 (0) | 0 (0) |  |  | 2 (50.0) | 0 (0) |  |
| 5 |  |  |  |  | |  |  |  |  |  |  | 1.000 |
| Remission | - | - |  |  | | - | - |  |  | 0 (0) | 0 (0) |  |
| Partial response | - | - |  |  | | - | - |  |  | 0 (0) | 0 (0) |  |
| No response | - | - |  |  | | - | - |  |  | 1 (100.0) | 1 (100.0) |  |

Data are presented as number (percentage); PSC, primary sclerosing cholangitis; IBD, inflammatory bowel disease.

**Supplementary Table 9.** Cohort demographics and disease characteristics among each treatment subgroup within the PSC-IBD group.

| Variables | IFX  (*n* = 19) | VDZ  (*n* = 28) | SMD/  *P*-value |  | IFX  (*n* = 19) | UST  (*n* = 21) | SMD/  *P*-value |  | VDZ  (*n* = 28) | UST  (*n* = 21) | SMD/  *P*-value |
| --- | --- | --- | --- | --- | --- | --- | --- | --- | --- | --- | --- |
| Age, years | 33 (26–42) | 38 (33.5–49) | 0.55 |  | 33 (26–42) | 38 (32–44) | 0.47 |  | 38 (33.5–49) | 38 (32–44) | 0.05 |
| Sex |  |  | 0.37 |  |  |  | 0.29 |  |  |  | 0.08 |
| Female | 8 (42.1) | 7 (25.0) |  |  | 8 (42.1) | 6 (28.6) |  |  | 7 (25.0) | 6 (28.6) |  |
| Male | 11 (57.9) | 21 (75.0) |  |  | 11 (57.9) | 15 (71.4) |  |  | 21 (75.0) | 15 (71.4) |  |
| Body mass index, kg/m^2^ | 21.7 (20.2–24.1) | 22.4 (20.3–25.0) | 0.41 |  | 21.7 (20.2–24.1) | 22.2 (20.9–23.4) | 0.24 |  | 22.4 (20.3–25.0) | 22.2 (20.9–23.4) | 0.17 |
| Smoker |  |  | 0.40 |  |  |  | 0.15 |  |  |  | 0.25 |
| Yes | 5 (26.3) | 3 (11.1) |  |  | 5 (26.3) | 4 (20.0) |  |  | 3 (11.1) | 4 (20.0) |  |
| No | 14 (73.7) | 24 (88.9) |  |  | 14 (73.7) | 16 (80.0) |  |  | 24 (88.9) | 16 (80.0) |  |
| CCI, mean ± standard deviation | 0.4 ± 0.8 | 0.6 ± 1.3 | 0.24 |  | 0.4 ± 0.8 | 0.6 ± 0.9 | 0.23 |  | 0.6 ± 1.3 | 0.6 ± 0.9 | 0.06 |
| IBD subtype |  |  | 0.65 |  |  |  | 0.18 |  |  |  | 0.46 |
| UC | 11 (57.9) | 24 (85.7) |  |  | 11 (57.9) | 14 (66.7) |  |  | 24 (85.7) | 14 (66.7) |  |
| CD | 8 (42.1) | 4 (14.3) |  |  | 8 (42.1) | 7 (33.3) |  |  | 4 (14.3) | 7 (33.3) |  |
| Age at initial diagnosis, years | 24 (16–30) | 23 (17.5–26.5) | 0.09 |  | 24 (16–30) | 23 (18–27) | 0.09 |  | 23 (17.5–26.5) | 23 (18–27) | 0.18 |
| Duration of IBD, years | 11 (6–13) | 16 (11.5–24) | 0.84 |  | 11 (6–13) | 13 (11–18) | 0.62 |  | 16 (11.5–24) | 13 (11–18) | 0.25 |
| Montreal classification for UC |  |  | 0.44* |  |  |  | 0.00* |  |  |  | 0.44* |
| Proctitis | 0 (0.0) | 1 (4.3) |  |  | 0 (0.0) | 0 (0.0) |  |  | 1 (4.3) | 0 (0.0) |  |
| Left-sided | 0 (0.0) | 1 (4.3) |  |  | 0 (0.0) | 0 (0.0) |  |  | 1 (4.3) | 0 (0.0) |  |
| Pancolitis | 11 (100.0) | 21 (91.3) |  |  | 11 (100.0) | 14 (100.0) |  |  | 21 (91.3) | 14 (100.0) |  |
| Montreal classification for CD |  |  | 0.35** |  |  |  | 0.14** |  |  |  | 0.49** |
| Ileal | 1 (12.5) | 0 (0.0) |  |  | 1 (12.5) | 1 (14.3) |  |  | 0 (0.0) | 1 (14.3) |  |
| Colon | 3 (37.5) | 2 (66.6) |  |  | 3 (37.5) | 2 (28.6) |  |  | 2 (66.6) | 2 (28.6) |  |
| Ileocolon | 4 (50.0) | 1 (33.3) |  |  | 4 (50.0) | 4 (57.1) |  |  | 1 (33.3) | 4 (57.1) |  |
| Primary outcome^a^ |  |  | 1.000  (Fisher’s exact test) |  |  |  | 0.027  (Fisher’s exact test) |  |  |  | 0.007  (Fisher’s exact test) |
| Remission | 13 (68.4) | 19 (67.9) |  |  | 13 (68.4) | 8 (38.1) |  |  | 19 (67.9) | 8 (38.1) |  |
| Partial response | 2 (10.5) | 4 (14.3) |  |  | 2 (10.5) | 1 (4.8) |  |  | 4 (14.3) | 1 (4.8) |  |
| No response | 4 (21.1) | 5 (17.9) |  |  | 4 (21.1) | 12 (57.1) |  |  | 5 (17.9) | 12 (57.1) |  |

Data are presented as median (interquartile range) or number (percentage), except CCI; UC, ulcerative colitis; CCI, Charlson Comorbidity Index (Modification Quan *et al.*); IBD, inflammatory bowel disease; CD, Crohn’s disease; IFX, infliximab; UST, ustekinumab; SMD, standardized mean difference;*, SMD calculated pancolitis vs no pancolitis; **, SMD calculated ileocolon vs no ileocolon.

^a^Clinical response was evaluated using partial Mayo Score (pMS) for UC and Harvey Bradshaw-Index (HBI) for CD: remission: pMS ≤ 1/HBI < 5; particular response: a decrease of pMS ≥ 2 or a decrease of HBI > 3; no-response.


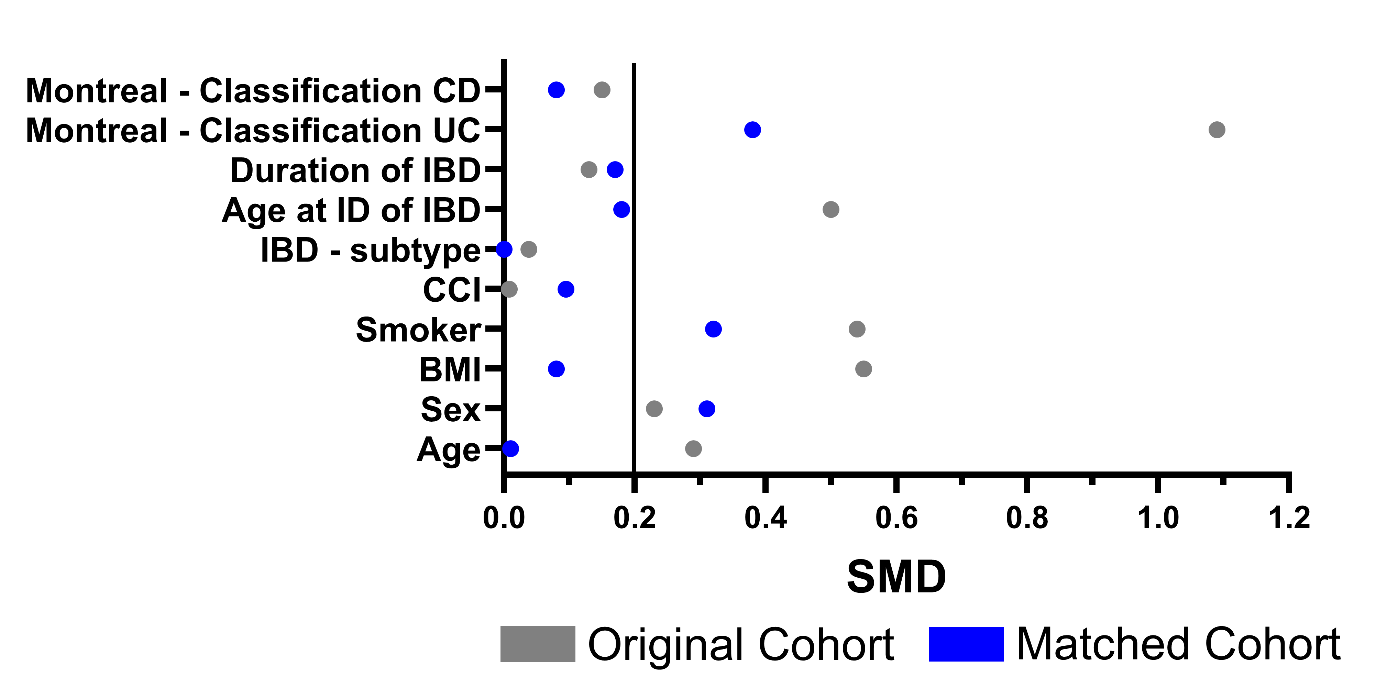


**Supplementary Figure 1.** Comparison of the SMDs between original and matched cohort in love plot. BMI, body mass index; CCI, Charlson Comorbidity Index; CD, Crohn’s disease; IBD, inflammatory bowel disease; ID, initial diagnosis; SMD, standardized mean difference; UC, ulcerative colitis.


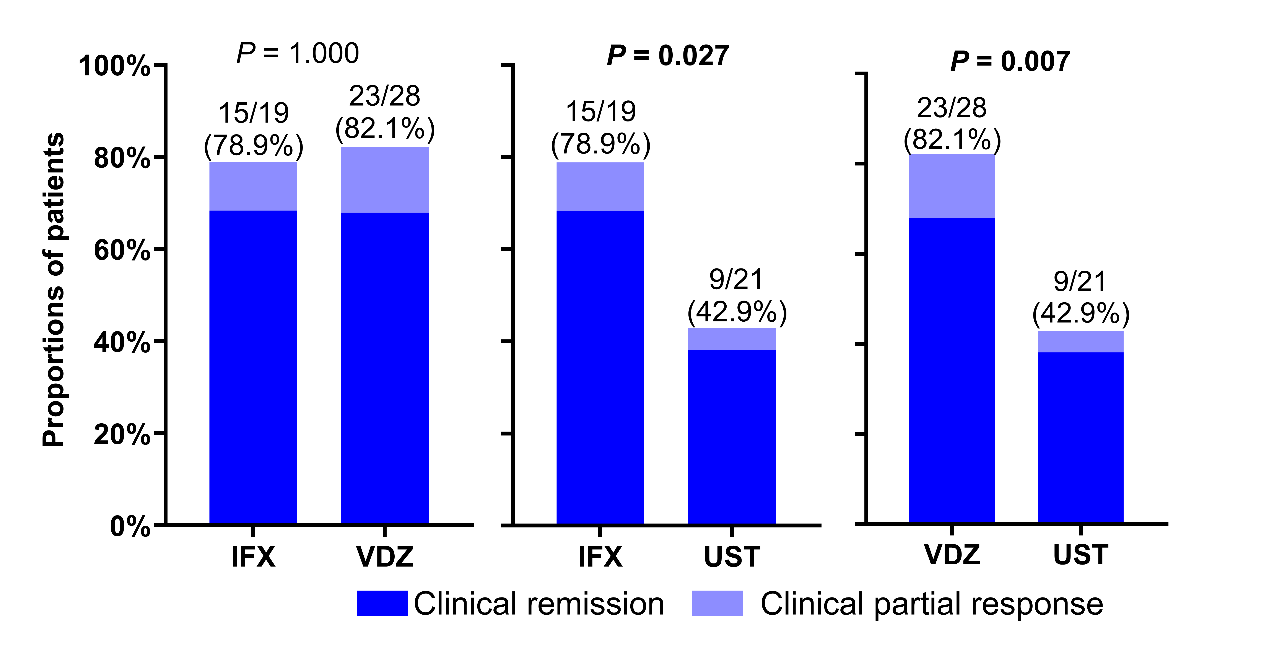


**Supplementary Figure 2**. Head-to-head comparison of the clinical therapy response within 20 weeks within the PSC-IBD group. IBD, inflammatory bowel disease; IFX, infliximab; PSC, primary sclerosing cholangitis; UST, ustekinumab; VDZ, vedolizumab.
